# A quantitative systematic review of the association between nurse skill mix and nursing‐sensitive patient outcomes in the acute care setting

**DOI:** 10.1111/jan.14194

**Published:** 2019-10-03

**Authors:** Diane E. Twigg, Yvonne Kutzer, Elisabeth Jacob, Karla Seaman

**Affiliations:** ^1^ School of Nursing and Midwifery Edith Cowan University Joondalup Western Australia Australia; ^2^ Centre for Nursing Research Sir Charles Gairdner Hospital Nedlands Western Australia Australia

**Keywords:** nurses, nursing outcomes, outcome assessment, outcomes (health care), outcomes research, patient outcome assessment, review, skill mix, systematic review, treatment outcome

## Abstract

**Aims:**

To examine the association between nurse skill mix (the proportion of total hours provided by Registered Nurses) and patient outcomes in acute care hospitals.

**Design:**

A quantitative systematic review included studies published in English between January 2000 – September 2018.

**Data sources:**

Cochrane Library, CINAHL Plus with Full Text, MEDLINE, Scopus, Web of Science and Joanna Briggs Institute were searched. Observational and experimental study designs were included. Mix‐methods designs were included if the quantitative component met the criteria.

**Review methods:**

The Systematic Review guidelines of the Joanna Briggs Institute and its critical appraisal instrument were used. An inverse association was determined when seventy‐five percent or more of studies with significant results found this association.

**Results:**

Sixty‐three articles were included. Twelve patient outcomes were inversely associated with nursing skill mix (i.e., higher nursing skill mix was significantly associated with improved patient outcomes). These were length of stay; ulcer, gastritis and upper gastrointestinal bleeds; acute myocardial infarction; restraint use; failure‐to‐rescue; pneumonia; sepsis; urinary tract infection; mortality/30‐day mortality; pressure injury; infections and shock/cardiac arrest/heart failure.

**Conclusion:**

Nursing skill mix affected 12 patient outcomes. However, further investigation using experimental or longitudinal study designs are required to establish causal relationships. Consensus on the definition of skill mix is required to enable more robust evaluation of the impact of changes in skill mix on patient outcomes.

**Impact:**

Skill mix is perhaps more important than the number of nurses in reducing adverse patient outcomes such as mortality and failure to rescue, albeit the optimal staffing profile remains elusive in workforce planning.

## Abstract (zh)

**目的:**

在于探讨护士技巧组合(注册护士提供的总时数比例)与急性护理医院患者结果之间的联系。

**设计:**

一项定量系统综述包括2000年1月至2018年9月期间以英语发表的各项研究。

**资料来源:**

搜索了Cochrane Library、CINAHL Plus with Full Text、MEDLINE、Scopus、Web of Science和Joanna Briggs Institute。纳入了观察性和实验性研究设计。如果定量成分符合标准,则纳入混合方法设计。

**综述方法:**

使用了Joanna Briggs Institute的系统综述指南及其关键评估工具。当75%或者更多具备显著结果的研究发现这种联系时,就确定了反向关联。

**结果:**

共纳入了63篇文章。12名患者的结果与护理技巧组合呈负相关(即,较高的护理技能组合与患者结果的改善显著相关)。这些是住院天数;溃疡、胃炎和上消化道出血;急性心肌梗死;约束使用;救援失败;肺炎;败血症;尿路感染;死亡率/30天死亡率;压力性损伤;感染和休克/心脏骤停/心力衰竭。

**结论:**

护理技巧组合影响了12例患者的结果。然而,需要使用实验性或纵向研究设计来进一步调查,从而建立因果关系。需要就技巧组合的定义达成共识,以便能够更有力地评估技巧组合变化对患者结果的影响。

**影响:**

在降低死亡率和救援失败等不良患者结果方面,技巧组合可能比护士人数更为重要,尽管最佳的人员配置在人力资源规划中仍然让人难懂。

## 1 INTRODUCTION

In their report in the year 2000, the World Health Organization highlighted both the importance and the challenges associated with finding the right mixture of healthcare personnel to achieve the best possible outcomes with the staffing resources available (World Health Organization, 2000). In this regard, consideration of the nursing workforce is critical. Skill mix, defined as ‘the proportion of total nurse hours provided by Registered Nurses’ (Twigg, Duffield, Bremner, Rapley, & Finn, 2012) (page 2,711), is an important element and involves assessing the mix of nursing staff, both registered and unregistered on a ward and varies in configuration dependent on the country where it is discussed. The nurse mix may include Registered Nurses (RNs), Enrolled Nurses, licensed practical nurses (LPNs), certified nursing assistants (CNAs), assistants in nursing (AINs), healthcare assistants, or other classifications (Jacob, McKenna, & D'Amore, 2015).

Various studies have since focussed on the importance of nurse staffing levels in an attempt to define an optimal configuration (Aiken, Clarke, & Sloane, 2002; Kim & Bae, 2018; Leary et al., 2016). Whilst research has explored staffing levels and their impact on patient outcomes, it does not always address nursing skill mix. A systematic review and meta‐analysis published in 2007 for example (Kane, Shamliyan, Mueller, Duval, & Wilt, 2007), which analysed nurse staffing levels, focussed on the ratio of Registered Nurses (RNs) to patients and patient outcomes, but not on nurse skill mix.

Despite these efforts, clear evidence‐based guidelines on staffing levels are lacking, particularly in regard to skill mix (Brennan, Daly, & Jones, 2013; Sharma, Hastings, Suter, & Bloom, 2016). A recent systematic review (Myers, Pugh, & Twigg, 2018) that examined the importance of nurse skill mix on patient outcomes focussed specifically on stand‐alone high acuity areas, meaning that findings from this review may not be easily transferrable to other care settings. The only other systematic review on skill mix and patient outcomes (Lankshear, Sheldon, & Maynard, 2005) examined articles published up to 2004 and highlighted the relationship between nurse staffing factors (Registered Nurse staffing levels and proportion of RNs in the skill mix) and patient outcomes. Their outcomes included mortality rates, complication rates (pneumonia, urinary tract infections, nosocomial infections, wound infections), failure‐to‐rescue, incidence of adverse events (falls, medication errors), length of stay, or patient satisfaction.

Of additional concern, continuing economic constraints and impending nursing shortages worldwide have led to the increased deployment of less qualified and unregulated health professionals, leading to changes in *skill mix* in the configuration of the nursing teams (Jacob et al., 2015; Roche, Duffield, Friedman, Dimitrelis, & Rowbotham, 2016). This is despite at least one study finding increases in unregulated workers has been associated with poorer patient outcomes (Twigg et al., 2016). With limited synthesis of the evidence to inform such staffing decisions, there is little guidance for policy makers and managers making those decisions.

The present review aims to expand on previous research by examining more recent studies up to 2018 exploring the impact of nurse skill mix on patient outcomes. The outcome of the review may help inform staffing policy in regard to skill mix changes.

### 1.1 Background

The conceptual framework developed by McCloskey and Diers (2005) was used to guide this review and the selection of variables. McCloskey and Diers (2005) examined the effects of health policy on nursing and patient outcomes sing the work of Aiken et al. (2002). McCloskey and Diers (2005) modified Aiken's framework to embed the seminal work of Donabedian's structure‐process‐outcomes framework (Donabedian, 1966). Structure was identified as nursing workforce characteristics such as nurse‐to‐patient ratios and skill mix. Process was identified as the processes of care. Outcome was identified as nurse and patient outcomes. This framework has been used to guide further studies including an examination of skill mix (Twigg, Duffield, Bremner, Rapley, & Finn, 2011; Twigg et al., 2012; Twigg, Gelder, & Myers, 2015). In this review, the proportion of total nurse hours provided by Registered Nurses was the structural variable and patient outcomes the outcome variable under review. It was hypothesized that changes in skill mix, for example, fewer Registered Nurse hours, affect the processes of care (such as recognition and response to patient changes) which in turn may impact on patient outcomes.

## 2 THE REVIEW

### 2.1 Aims

The aim of the review was to synthesize the available quantitative evidence on the association of nursing skill mix and patient outcomes sensitive to nursing care in adult patients in acute care hospitals. As such, the review question was: What effect does skill mix have on nurse sensitive patient outcomes?

### 2.2 Design

The review used a quantitative systematic literature review. Methods of the analysis and inclusion and exclusion criteria were identified in advance and documented in a research protocol. The PICOS framework (Population; Intervention; Comparator; Outcome; Study design) (Schardt, Adams, Owens, Keitz, & Fontelo, 2007) was used to refine the inclusion and exclusion criteria and processes and outcomes were guided by the Joanna Brigg Institute for Systematic Reviews. Table 1 outlines the PICOS framework.

**Table 1:** PICOS framework

PICOS	Inclusion criteria	Exclusion criteria
Population:	Adult patients (aged over 18 years) in acute care hospital wards. No restrictions were imposed regarding hospital or ward size, teaching status or sector.	Participants in peri‐operative, maternity, paediatrics (aged < 18 years), mental health, substance abuse, nursing home or palliative care environments
Intervention:	Studies reviewing a particular type of nursing skill mix or skill mix level and/or compared them with a different (e.g., baseline) skill mix type or level were included in the review.	Studies examining patient‐to‐nurse ratios were excluded, as ratios had been covered by another review.
Comparator:	A different nursing skill mix level or no comparator.	
Outcome:	Patient outcomes (sensitive to nursing care) such as mortality, deep vein thrombosis, sepsis, urinary tract infection, pressure injuries, pneumonia, upper gastro‐intestinal bleeding, shock/cardiac arrest, central nervous system complications, surgical wound infections, pulmonary failure, physiologic/metabolic derangement, or any others identified (and tested) by researchers as outcomes potentially sensitive to nursing care	
Study design:	Observational/descriptive (includes cross‐sectional, prospective/cohort studies, case‐control studies) and experimental (includes experimental [RCT], quasi‐experimental [time‐series etc.]), and mixed methods designs were included if the quantitative component was relevant to the research question.	Qualitative studies

Relevant patient outcomes were identified by reviewing existing literature on the topic (Aiken et al., 2014; Duffield et al., 2011; Needleman, Buerhaus, Mattke, Stewart, & Zelevinsky, 2002; Twigg et al., 2011). The review focussed on acute care hospitals and included studies set in general medical, surgical, combined medical/surgical and step‐down wards, telemetry units and emergency departments. Acute care settings were chosen as the purpose of this review was to expand on previous studies by specifically examining the importance of skill mix in optimal staffing and previous studies were primarily undertaken in acute care settings. Nurse‐reported patient outcomes were not included in the study as the outcome was not directly measured. Exclusion criteria were applied as listed in Table 1.

### 2.3 Search methods

Prior to commencing the review, searches were run in Medline and CINAHL to identify whether similar systematic reviews had already been completed in the last 10 years. The PROSPERO register was also checked to determine whether similar reviews were already underway. One study was found to be similar and at the initial protocol development stage, the corresponding author was contacted to determine the progress of the review; however; there was no response provided nor was any output from the systematic review published. A copy of the protocol for this study is provided in the Data S1 (Skill Mix OnlineSUPP_A_SystReviewProtocol).

Studies were identified through searching electronic databases, hand searching the reference lists of relevant articles, author searches, and via examining grey literature databases. In addition, an EBSCOhost Alert Notification and a Web of Science Search Alert was set up to capture any new publications between September 2016 and June 2017. An updated search strategy was conducted in September 2018. A health information librarian was consulted during the development of the MeSH terms (nursing assistants; mortality; sepsis; urinary tract infection; pneumonia; clinical deterioration; quality of health care; patient safety; accidental falls; surgical wound infection; venous thrombosis; shock; heart arrest; medication errors; infection; central nervous system infections). The search strategy was applied to Medline and CINAHL Plus with Full Text and was adapted for Embase, the Cochrane Library, Scopus, Web of Science and the Joanna Briggs Institute Database. The search strategy for each database is outlined in the Data S2 (Skill Mix OnlineSUPP_B_ElectDatabaseSearchStrat.pdf). Grey literature catalogues used included Research Online, Open Grey/EThOS, OAIster, Google Advanced Search and PsycEXTRA.

Limiters included articles published between January 2000 – September 2018 and written in English.

### 2.4 Search outcome

The search strategy identified a total of 2,702 citations from electronic databases. A comprehensive search for grey literature and hand searches produced an additional 12 articles. After removing duplicates, 2,576 articles were retained and their titles and abstracts subsequently screened for inclusion. This process led to 2,481 citations being excluded and the full‐text of the 95 remaining publications was assessed for eligibility.

### 2.5 Quality appraisal

The critical appraisal of the selected 95 records was undertaken using the Joanna Briggs Institute (JBI) critical appraisal tools (Godfrey & Harrison, 2015). All studies were examined independently by two reviewers, and the results of the critical appraisal reviewed after both reviewers had completed their assessment and discrepancies resolved by consensus.

**Figure 1:**
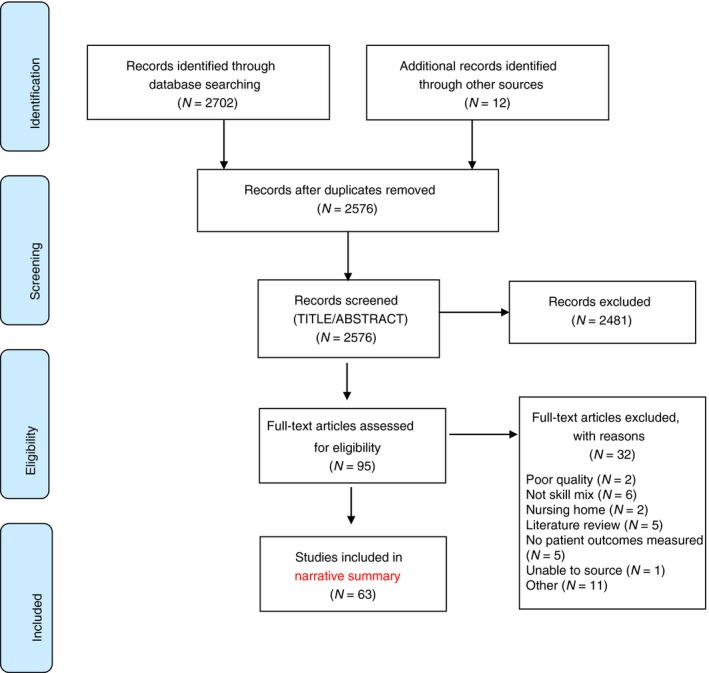
Skill mix systematic review prisma flow diagram [Colour figure can be viewed at http://www.wileyonlinelibrary.com]

Thirty‐two articles were excluded following the full‐text assessment and are detailed further in Data S3 (Skill Mix OnlineSUPP_C1‐4_CritAppraisals.pdf). Of these, three studies were excluded as they were deemed to be of insufficient quality (no reported data, unequal comparison group, small sample size); six did not review any skill mix variables and a further five did not measure any nursing‐sensitive patient outcomes; two studies were conducted in a nursing home setting and therefore did not meet inclusion criteria; five studies were literature reviews, three articles could not be sourced and an additional eight articles were excluded for other reasons (not primary research articles, measured nurse perception, cost analysis). This left 63 articles, which were included in the narrative summary. A record of study assignment was kept, which contained the reviewing authors recommendations about inclusion or exclusion as well as some comments on why an article was retained or rejected. Figure 1 outlines the study flow chart and the articles selected and excluded at each step of the review. The critical appraisal scores for each of the 95 appraised studies are outlined in Data S3 (Skill Mix OnlineSUPP_C1‐4_CritAppraisals.pdf).

### 2.6 Data abstraction

One author conducted the initial extraction of the data from the selected studies and a second author checked for accuracy. It was planned that disagreements would be resolved via discussion between the two authors; if no agreement was reached, a third author would be consulted. Information was extracted from the included studies about country, population, setting and sample size, aim, study design, number of participants, type of skill mix variable measured, patient outcomes and general findings. The included articles were treated as equal in this study and are summarized in Data S4 (Skill Mix OnlineSUPP_D_IncStudyCharact.pdf) and include estimates of precision for each outcome. An abbreviated summary is reported in Table 2.

**Table 2:** Included study characteristics (a summary)

First author (year)	Setting	Aim	Study design; No. of participants	Definition of skill mix; comparison group	Patient outcomes	General findings
Aiken et al. (2016)	Acute care hospitals	To determine association of SM with mortality/ratings of care/quality indicators	Cross‐sectional; 13,077 nurses 275,519 Pts	% RN among total nsg personnel	Pt mortality; Pt ratings of care	Richer nsg SM associated with ↓ odds of mortality, ↓ odds of reports of poor quality, poor safety grades,
Ambrosi et al. (2017)	Acute internal medicine	In‐hospital mortality rates	Secondary analysis longitudinal observational study; *N* = 1,464	% care offered from RNs.	Mortality (% died in hospital)	Pts more at risk of dying at weekends. Pts receiving a higher SM were at less risk of dying
Anthony (2008)	Medical‐surgical units	What is relationship of RN staffing & adherence to practice guidelines	Retrospective correlational design; *N* = 210	Total nursing department HPPD, & proportion of RNs	Total number of episodes of hypoglycaemia	SM for Hospital A was positively related for hypoglycaemic patients, but not for Hospital B.
Aydin et al. (2015)	Medical‐surgical units	The impact of nsg on falls, injury from falls, & restraint.	Multivariate study testing predictive models; *N* = 789 Units	% RN HPPD; % LVN HPPD; % un‐licensed HPPD; % Sitter hours	Falls and injury falls; restraint	↑ SM resulted in improvement in Pt safety and injuries. ↓ falls/injury predicted by ↑ unlicensed care hours.
Bae, Kelly, Brewer, and Spencer (2014)	Acute care units	Explore association b/w nsg staffing characteristics and Pt falls/PU.	Retrospective observational design *N* = 35 Hospital Units	Proportion of RNs to LPNs and UAPs	Pt falls; Pt falls injuries; PU	Pt falls & injury falls ↑ with ↑ temporary RN staffing levels, but ↓ with ↑ levels of LPN care HPPD.
Ball et al. (2018)	Acute care hospitals	Explore association b/w nsg staffing levels and mortality and missed care.	Retrospective cohort study; *N* = 422,730	RN/Pt ratio, Nurse Education; % with degree	30‐day in‐Pt mortality	Each additional Pt/nurse associated with 7% ↑ in odds of Pt dying ≤30 days of admission.
Barkell, Killinger, and Schultz (2002)	Surgical unit	Examine effects of changed staffing model on LOS, cost, Pt satisfaction	Retrospective, descriptive comparison design. *N* = 59 (model A); 37 (model B)	% of RNs of total caregiver staff	LOS; Pnem; UTI; pain	Pts’ perception of pain was statistically significant. Mean number of pain scores ↓ slightly in higher SM
Blegen, Goode, Spetz, Vaughn, and Park (2011)	General and ICUs	Determine relationship b/w Pt outcomes, staffing and safety net status	Cross sectional administrative datasets; 1.1 million adults	% of hrs provided by RNs.	CHF mortality; PU; infections; FTR; Post op sepsis; LOS	RN SM in general units was associated with ↓ FTR and infections, and in ICU with fewer cases of sepsis and FTR.
Bolton et al. (2007)	Medical‐surgical units	What is the impact of nsg ratios on nursing quality?	Follow‐up analysis; *N* = 252 Units	No definition supplied	Falls; PU	Significant −ve associations between % of contracted staff & falls with injury; % of care hrs by RN staff and falls.
Boyle et al. (2016)	Patient care units	Develop a unit‐level inpt composite nsg care quality performance index	Two‐phase measure development study; *N* = 5,144 Hospital Units	Total nsg HPPD and nsg SM	PU and Fall Rate Quality Composite Index	Nsg HPPD, RN SM, RNs with degrees or specialty certification, agency nurses hrs significantly associated with PUFRQCI.
Breckenridge sproat, Johantgen, and Patrician (2012)	Army hospital	What are the associations of nurse staffing and workload on Army hospital units?	Secondary analysis—longitudinal data set; *N* = 23 Hospital Units	% total nsg care hrs worked each shift by RNs/LPNs/NAs.	Medication errors, falls	A ↑ % of LPNs associated with ↑ medication error rate but RN SM not a statistically significant predictor.
Chang & Mark (2011)	Medical‐surgical	Explore relationship b/w learning climate and medication errors	Cross‐sectional descriptive study; *N* = 2,744	% nsg care hours delivered by RNs; all nsg personnel	Medication errors	When learning climate was −ve, having more RNs was associated with ↓ medication errors.
Cho, Ketefian, Barkauskas, and Smith (2003)	Acute care hospitals	What are the effects of nsg staffing on adverse events, morbidity, mortality, costs?	Cross‐sectional descriptive study *N* = 124,204	RN proportion + RN hrs divided by all hrs	Pt fall, PU, Pnem, ADE, UTI, sepsis, wound infection	Significant inverse relationship between RN hrs, RN % and pnem (ie ↑ of 1 RN hr = 8.9% ↓ chance of pnem.
Choi & Staggs (2014)	Acute care units	What is the relationship between 6 nsg staffing measures and UAPUs?	Descriptive, correlational study; *N* = 57,223 RNs	% of total RN nsg hrs; total nsg HPPD and non‐RN HPPD.	UAPUs	A ↑ of 1% point in RN SM was associated with an estimated 1.2% ↓ in UAPU odds.
de Cordova, Phibbs, Schmitt, and Stone (2014)	VA hospital units.	Explore relationship b/w RN levels, SM, and experience on night shift to LOS?	Longitudinal descriptive study; *N* = 8,243	SM day shift %, compared to each group.	LOS	% HPPD provided by UAPs and presence of larger % UAPs in relation to RNs associated with ↑ LOS.
Donaldson et al. (2005)	Acute care Hospitals	What is the impact of minimum‐staffing ratios on nsg hours and SM?	Cross‐sectional study; Total pt days: Approximately 196,000	% RN hours; % LVN nursing‐care hours	Pt falls, PU	No significant changes were found despite research linking nurse staffing with fall rates and PU.
Duffield et al. (2011)	Acute care units.	Relationship between work environment, workload, nsg SM, & pt outcomes	Longitudinal retrospective & cross‐sectional; payroll records *N* = 10,963,806	% of RNs on unit; % of CNSs, ENs, AINs, and TENs.	11 NSO	An ↑ in RN/CNS hours was associated with significant ↓ in 6 NSO
Esparza, Zoller, White, and Highfield (2012)	acute care hospitals	What is relationship b/w RN staffing and SM patterns?	Cross‐sectional study; Over 2 million pt discharges analysed	% RN hours, mandated nurse‐pt ratios	UTI; LOS	As RN % of SM ↑, OR of UTI ↓x4.25. ↑ % of RN skill mix = shorter LOS.
Estabrooks, Midodzi, Cummings, Ricker, and Giovannetti (2005)	Acute care hospitals	What is effect of nsg characteristics on 30‐day hospital mortality rate?	Cross‐sectional analysis; *N* = 18,142 pts	RN; total nsg staff; non‐RN staff.	30‐day pt mortality	Hospitals with a higher proportion of RNs were associated with ↓ rates of 30‐day pt mortality.
Frith et al. (2010)	Medical‐surgical units.	What are the effects of nsg staffing on hospital‐conditions and LOS?	Cross‐sectional retrospective study *N* = 34,838 pt cases	% RN staff on units, compared to % LPNs.	LOS; adverse events	Both RN and LPN % were significantly − ve related to ↓ LOS. ↑ % RNs in SM predicted ↓ adverse events and ↓ LOS.
Glance et al. (2012)	Trauma centres	Association between nsg staffing and hospital outcomes in injured pts?	Cross‐sectional study; *N* = 70,142 pts	No definition supplied. Change in LPN staffing levels	Mortality; FTR; infections	1% ↑ in ratio of LPN to total nsg time associated with 4% ↑ in odds of mortality and 6% ↑ in odds of sepsis.
Goode, Blegen, Park, Vaughn, and Spetz (2011)	Magnet/Non‐Magnet hospitals	What is relationship between staffing and pt outcomes?	Bivariate and multivariate analyses. *N* = 54 Hospitals	RN staffing mix % at Magnet/Non‐Magnet hospitals.	Mortality; FTR; HAPU; infections; postop sepsis; LOS	Non‐Magnet hospitals had 2% ↑ RN SM than Magnet hospitals.
He, Staggs, Bergquist‐Beringer, and Dunton (2016)	NDNQI hospitals	To identify longitudinal relationship between nurse staffing and pt outcomes.	Longitudinal study *N* = 13,339 total falls, *N* = 12,435 PU	RN SM compared to total nsg HPPD by all staff.	Falls; HAPU	RN SM positively associated with fall rate, inversely associated with rate of PU (stage III or above).
He, Almenoff, Keighley, and Yu‐Fang (2013)	VA medical centres	Assess impact of pt‐level risk adjustment on associations of nsg staffing and mortality	Retrospective cross‐sectional study; *N* = 284,097 pts	Total RN hours compared to total nsg productive HPPD.	30‐day inpatient mortality	For non‐ICU, ↑ RN SM was associated with ↓ mortality risk.
Huston (2001)	Surgical units;	Identify correlations between changing staffing mix and postop pain mgt?	Retrospective descriptive study. *N* = 203 pts	% RNs with direct pt care responsibility.	Pain	Relationship identified between mean pain scale scores and UAP staffing versus RN staffing.
Johansen, Cordova, Duan, Martinez, and Cimiotti (2015)	ED	What is the effect of nsg resources on the process of care in ED?	Secondary analysis of ED data. Hospitals: 73 Patients: 1,343	RNs, LPNs, and aides	Care processes for ACS or acute MI	Each 10% ↑ in proportion of RNs associated with a 7.1% ↑ in aspirin on arrival and a 6.3% ↓ in PCI in timeframes
Kim, Park, Han, Kim, and Kim (2016)	Acute care hospital	Evaluate the effects of nsg staffing on hospital readmission of COPD pts	Retrospective observational study Hospitals: 1,070	Number of RNs per 100 beds; proportion of RNs on staff.	Readmission to hospital within 30 days	A ↑ proportion of RNs was significantly associated with a ↓ readmission rate.
Kim, Park, et al. (2016)	Acute care hospitals	Explore relationship b/w nsg levels, LOS and expenses of hip/knee surgical pts	Cross‐sectional study Hospitals: 222, Pts: 22,289	Bed to RN ratio; bed to NA ratio; nsg staff grade; % RNs	LOS of hip/knee surgery pts	Each ↑ number of beds per RN = ↑ LOS by 0.7 days. Median or higher bed‐to‐nurse ratio had an ↑ LOS of 4.89 days.
Kim & Han (2018)	Tertiary hospitals	Explore the relationship b/w nsg level with NSO	Retrospective observational study Hospitals: 46	Grades determined by the ratio of beds to RNs	12 NSOs	Statistically significant associations between higher nurse staffing level and rates for all NSOs except PU.
Lake, Shang, Klaus, and Dunton (2010)	Magnet and non‐Magnet hospitals	What is the relationship b/w nsg unit staffing, Magnet status, and pt falls?	Retrospective cross‐sectional observational study. Hospitals: 636	RN HPPD, compared to non‐RN HPPD LPN & NA	Pt falls	RN HPPD is −ve associated with fall rate; conversely LPN and NA HPPD were positively associated with fall rate.
Leary et al. (2016)	Acute care hospital	Explore relationship b/w RN and other nurse staffing levels and clinical outcomes	Descriptive correlational design. Hospital: 2 Units: 33	Staffing levels for RNs and HCSW	Falls; PU	Wards with a ↑ ratio of RN to HCSW have less falls. No significant correlation b/w staffing and PU.
Lee, Yeh, Chen, and Lien (2005)	General hospital	Examine personnel cost and quality of care after implementing the SM practice model.	Pre‐ and post‐test quasi‐experimental design Hospitals: 1	SM practice model. Nurses and NAs.	Falls; medication error rate	Fall rate and medication error rate showed no statistically significant variation.
Martsolf et al. (2014)	Acute care hospitals	Effect of nurse staffing on quality of care and inpatient care costs.	Retrospective longitudinal study; Hospitals: 421 *N* = 18,474,860	Total nursing staff (licensed + aides) per 1,000 patient days	LOS; adverse events	↑ nurse staffing levels was associated with reduced adverse events and LOS. ↑ RNs associated with ↓ patient care costs.
McCloskey & Diers (2005)	Acute public hospitals	Examine effects of hospital reengineering on adverse pt outcomes.	Retrospective longitudinal study; Hospitals: 85 *N* = 3.3 million discharges	% of total nursing FTEs who were RNs	11 NSOs	Substantial ↑ in many adverse clinical outcomes after reengineering's implementation.
McGillis Hall and Doran (2004)	Acute hospital units	Assess the effect of different nurse staffing models on costs and pt outcomes	Descriptive correlational design; Hospitals: 19 Units: 77	Variations of SM including RN/RPN/UAP	Patient falls; UTIs; medication errors; wound infections.	Lower proportions of professional nursing staff associated with ↑ ion errors and wound infections.
McGillis Hall, Doran, and Pink (2004)	Acute hospital units	Explore staffing models and demographic variables effect on pt outcomes.	Descriptive correlational design; Hospitals: 19 Units: 77	RN/RPN/UAP mix.	Patient falls; UTIs medication errors; wound infections;	All RN staff model had statistically significant +ve relationship on nurses’ perceptions of quality of care.
McGillis Hall et al. (2003)	Teaching hospitals	Evaluate the impact of different nurse staffing models on the pt outcomes	Repeated‐measures study Hospitals: 19, *N* = 2046	Nurse staff‐mix included all RN, RPN, and UAP.	Change pain control	SM of RNs and UAP associated with better pain outcomes at discharge than a SM of RNs/RPNs and UAP.
Needleman et al. (2002)	Non‐federal hospitals	Examined the relation b/w the level of nsg staff and rate of adverse outcomes.	Descriptive correlational design. Hospitals: 799	Hrs of care by licensed nurses (RN–hrs plus LPN–hrs)	14 NSOs	A ↑ proportion of hours of nsg care provided by RNs resulted in 3%–5% shorter LOS and ↓ 2%–9% complications.
Newhouse et al. (2013)	Acute care hospitals	Evaluation of a rural hospital quality collaborative and organisational context	RCT ‐ crossover. Hospitals: 23	Hrs worked by each type of nsg staff	Heart failure core measures	SM associated with no statistically significant changes during intervention period on all 4 core measures.
Park, Blegen, Spetz, Chapman, and Groot (2012)	Non‐ICU units	Examined relationship b/w RN staffing and FTR carried with pt turnover levels.	Descriptive correlational design; Hospitals: 42 Units: 759	RN HPPD	FTR	Higher RN staffing levels on non‐ICUs were significantly associated with lower rates of FTR.
Patrician et al. (2011)	Military hospitals	The association between nurse staffing and adverse events at the shift level.	Longitudinal, correlational; Hospitals: 13, Units:56	Hrs worked by RNs, LPNs, & unlicensed providers.	Patient falls; medication errors	Greater proportion of RNs significantly associated with fewer falls and less medication errors some wards.
Patrician et al. (2016)	Military hospitals	Evaluate the effects of nurse staffing on HAPU development	Longitudinal, correlational Hospitals: 13 Units: 56, *N* = 1,643	% RN, % LPN, % NA	HAPU development	RN SM was not associated with HAPU. ↓ levels of total nsg care was associated with HAPU
Paulson (2004)	Military hospital EDs	Wait time and no. of pts who LWBS using nurses verses UAP.	Comparative descriptive, retrospective chart review;	RN with associate's degree, LPN, RN with baccalaureate, c/w UAP.	Wait time of patients who LWBS	The average difference in pt wait time was 73 min (57% ↓; *p* < .000).
Person et al. (2004)	Project linked hospitals	Assess the association of nsg staff with in‐hospital mortality for pts with AMI.	Descriptive correlational design; Hospitals: 4,401 *N* = 118,940	FTE RNs; FTE LPN; average daily census	In‐hospital mortality for pts with AMI	↑ RN staffing associated with pts less likely to die. With higher LPN staffing, pts more likely to die in‐hospital.
Pitkaaho, Partanen, Miettinen, and Vehvilainen‐Julkunen (2015)	Acute care wards	Analyse relationships b/w nurse staffing and patients' LOS.	Retrospective longitudinal design; Hospitals: 1 *N* = 35,306 patient episodes	Average proportion of RNs.	LOS	RNs proportions of 65%–80% was conducive to ↓ LOS. Higher and lower % of RNs predicted ↓ likelihood of ↓ LOS.
Potter, Barr, McSweeney, and Sledge (2003)	Acute inpatient units	To examine the relationship of nurse staffing to pt outcome measures	Prospective, correlational design; Hospitals: 1 Units: 32	Average % of RN and UAP hrs of direct care	Patient falls; medication errors	No findings relating to SM. Higher number of care hr, irrespective of category, associated with fewer falls.
Roche, Duffield, Aisbett, Diers, and Stasa (2012)	Public hospitals	Examine the relationship between staffing, SM and incidence of NSOs	Longitudinal, descriptive; Hospitals: 2 Units: 14	RN hours as a % of total nsg hrs	7 NSOs	Increase of 10% in proportion of hours worked by RNs linked to ↓ in NSO rates.
Schneider & Geraedts (2016)	Acute care hospitals	Association between nurse and physician staffing and the incidence of HAPU.	Cross‐sectional Hospitals: 720	% of nurses with 3 years of training; total nsg staff	Standardised incidence ratios of HAPU.	A 10% ↑ in the proportion of nurses with at least 3 years of training to total nsg staff was associated with a ↓ in HAPU.
Schreuders, Bremner, Geelhoed, and Finn (2015)	Tertiary hospitals	Examine the impact of nurse staffing on inpatient complications	Retrospective longitudinal hospitalization‐level study Hospitals: 3	Proportion of total nsg hours worked by RNs.	8 NSO	Direction of the association between nurse staffing and pt complications was not consistent across NSOs.
Seago, Williamson, and Atwood (2006)	Teaching hospital	Compare the relationship between nsg staffing and positive pt outcomes.	Longitudinal, retrospective repeated measures design Hospital: 1, Units: 3	Proportion of RN hours divided by total hours,	FTR from medication error; FTR from PU	There was an ↑ in FTR from medication error as the non‐RN hours of care per pt day increased. FTR from PU ↑ as SM ↑.
Sochalski, Konetzka, Zhu, and Volpp (2008)	Acute care hospitals	Explore whether ↑ in licensed nsg staff is associated with NSO	Cross‐sectional Acute MI *N* = 348,720 FTR *N* = 109,066	RN and RN/LVN nurse staffing	30‐day Acute MI mortality; surgical FTR	An increase in RN and RN ‐ LVN hours per pt day was not associated with reductions in acute MI mortality or FTR.
Sovie & Jawad (2001)	Teaching hospitals	Describe the effects of nsg structure and processes on selected pt outcomes	Descriptive, longitudinal Hospitals: 29	HPPD for all staff, for RN, UAP and Other	Fall rate; PU; UTI	Fall rate declines as number of RN HPPD increases.
Staggs & Dunton (2014)	NDNQI hospitals	Explore association b/w level of RN and non‐RN staffing and unassisted falls	Cross‐sectional, Hospitals: 1,361	RN HPPD & Non‐RN HPPD	Monthly unit‐level data on inpatient falls	For all unit types except rehabilitation, higher non‐RN staffing was associated with ↑ rates of unassisted falls.
Staggs, Knight, and Dunton (2012)	Hospitals using NDNQI	To explore hospital & nsg unit characteristics as predictors of fall rates	Longitudinal Hospitals: 248 Units: 1504	Proportion of total nsg care hrs provided by RNs.	Unassisted fall rate	↑ in proportion of nsg care hrs provided by RNs is associated with an estimated 4.0% average ↓ unassisted falls.
Staggs, Olds, Cramer, and Shorr (2016)	Hospitals using NDNQI	Examining whether nsg staff is associated with restraint use	Longitudinal Units: 3,101	Proportion of nsg hrs provided by RNs, no restraint used.	Reported restraint	Statistically significant effects of SM category on odds of any restraint and odds of fall prevention restraint.
Tourangeau, Giovannetti, Tu, and Wood (2002)	Acute care hospitals	Examine the effects that nsg care has on common quality of care outcomes	Retrospective design Hospitals: 75 Pt records (*N* = 46,941)	RN earned hrs c/w other nsg staff earned hrs	30 day risk‐adjusted mortality rate	10% increase in RNs associated with 5 fewer patient deaths per 1,000 discharges.
Twigg et al. (2016)	Acute care hospitals	Examine the impact of adding AIN to acute hospital ward staff on pt outcomes.	Descriptive cohort study Hospitals: 11 *N* = 256,302	NHPPD ratings for AIN wards c/w non‐AIN wards	7 NSOs	3 significant ↑ in adverse outcomes on the wards with AINs (FTR, UTI, falls with injury).
Twigg et al. (2012)	Multi‐day wards	Examine the association b/w SM and NSOs	Retrospective, longitudinal analysis. Hospitals: 3. Pt records (*N* = 103,330)	Proportion of total nurse hours provided by RNs (in %)	14 NSOs	↑ in SM associated with ↓ in the rates of 8 NSOs. There were significantly ↑ rates of 3 NSOs.
Tzeng, Hu, and Yin (2011)	Acute care hospitals	To determine two nsg staff indicators on the hospital‐acquired injurious fall rates.	Retrospective analysis Hospitals: 244	Precent of RN FTEs by total nsg personnel FTEs	Hospital‐acquired injurious fall rates	Higher % of RN FTEs by total nsg personnel FTEs did not result in decreased injurious fall rates.
Unruh (2003)	Acute care hospitals	To examine the relationship of licensed nursing staff with pt adverse events	Retrospective, longitudinal analysis. Hospitals: 211	Proportion of licensed nurses/total nsg staff	6 pt outcomes	Number of Licensed nurses both positively and negatively related to pt outcomes.
Unruh & Zhang (2012)	Acute care hospitals	To examine the relationship b/w changes in RN staffing and pt safety	Retrospective, longitudinal analysis. Hospitals: 124	RN FTEs and RN per adjusted patient day	PU; FTR; selected infections; Post op sepsis	RN FTEs positively and negatively related to NSOs.
Yang, Hung, and Chen (2015)	Respiratory care centre	Explore the impact of nsg staff models on pt safety, quality of care and costs.	Retrospective cohort study; *N* = 667	% of RNs to total nsg staff; 3 mixed models of nsg staffing,	8 NSOs	Different outcomes found b/w groups for medication errors, UTIs bloodstream infections and rate of ventilator weaning
Yang (2003)	Medical‐surgical units	Examine the relationship b/w hospital nurse staffing and pt NSOs	Retrospective, descriptive correlational design Units: 21, *N* = 29,424	Ratio of RNs to average pt.	Falls, PU, respiratory tract infections and UTIs.	Ratio of RNs to patient census negatively correlated to patient falls, UTI and complaints.

Abbreviations: ACS, acute coronary syndrome; ADE, adverse drug event; AIN, Assistants in Nursing; b/w, between; CHF, congestive heart failure; CNS, Clinical Nurse Specialist; COPD, chronic obstructive pulmonary disease; ED, Emergency department; EN, Enrolled Nurse; FTE, full time equivalent; FTR, Failure to rescue; HAPU, Hospital acquired pressure ulcers; HCSW, Healthcare Support Workers; HPPD, hours per patient day; Hr, hours; ICU, Intensive care unit; LOS, Length of stay; LPN, Licensed practical nurse; LVN, licensed vocational nurse; LWBS, left without being seen; Mgt, management; MI, myocardial infraction; *N*, number; NA, Nursing assistant; NDNQI, National Database of Nursing Quality Indicators; Nsg, nursing; NSO, nurse sensitive outcomes; OR, odds ratio; PUFRQCI, PU and Fall Rate Quality Composite Index; Pt, patient; Pnem, pneumonia; Post op, Post‐operative; PU, pressure ulcer; RPN, registered practical nurse; RN, Registered Nurse; SM, skill mix; TEN, Trainee Enrolled Nurses; UAP, unlicensed assistive personnel; UAPU, Unit acquired pressure ulcer; UTI, urinary tract infection; VA, Veteran Affairs; %, Percent; ↑, Increase/higher; ↓, Decrease/lower; −ve, negative; +ve, positive.

### 2.7 Synthesis

The included studies showed great variation as to how nurse skill mix was measured and conceptualized. They also used a variety of different study designs, making it not feasible to perform a meta‐analysis. The data were thus summarized narratively, comparing results where appropriate.

## 3 RESULTS

### 3.1 Settings

Most studies were completed in general acute care settings, including general medical, general surgical, combined medical/surgical, step‐down, telemetry units, and emergency departments in public hospitals. One study was conducted in a non‐federal hospital and four in veteran affairs or military hospital settings. Two studies were conducted in a respiratory care centre and trauma centre, respectively. Thirty‐six studies were assessed at unit level, 23 at hospital‐level, and four at shift level. Most studies were conducted in North America—40 in the USA and five in Canada. Another five studies were conducted in Australia, three in Taiwan and South Korea, one in New Zealand, Italy, UK, Germany and Finland, and two studies were conducted in Europe involving multiple countries.

### 3.2 Study designs

The studies included in this review were mainly observational studies without a comparison group. Thirty‐eight studies fell into this category. Another 14 studies were cross‐sectional, seven used a cohort study design with a comparison group, one was a quasi‐experimental design, one was a randomized controlled trial, one was descriptive correlations, and one was a two‐phase measure development study. The study designs of the included papers were generally low. There was one randomized control trial and two pre‐test–posttest quasi‐experimental studies. The remaining studies were either an observational study, analytic design or an observational descriptive study.

### 3.3 Nursing skill mix variables

The included studies used a variety of methods to define and assess skill mix. The most frequently used variable to measure nurse skill mix was ‘*percentage of nursing hours provided by RNs*’. Nineteen studies used this measure, defining skill mix either as ‘RN hours provided per day’, ‘RN hours provided per patient day’, ‘RN hours provided per shift, ‘number of productive hours worked by RNs’, or ‘RN hours provided per inpatient earned hours’. The ‘*percentage of RNs on staff*’ was also a commonly used way to define skill mix (16 studies). Percentage of RNs on staff was defined as ‘RN full‐time equivalent (FTE)’, ‘percentage of total RN nursing full‐time positions’, ‘RNs on the unit’, ‘percentage of professional nurses’, ‘proportion of licensed nurses (RN & Licensed practicing nurses (LPN))’, ‘percentage of RN on staff with direct caring responsibilities’ and ‘ratio of FTE RNs to average daily census’. Additional skill mix variables used for analysis included ‘nursing skill mix excluding assistants in nursing (AINs) (compared with skill mix including AINs)’, ‘the number of licensed nurses (RNs & LPNs) per 1,000 patient days’, ‘proportion of all licensed nurses (RN or LPN)’, ‘percentage of nurses with a minimum of three years training’, ‘proportion of regulated workers’, ‘mandated nurse ratios’, ‘triage systems using nurses only (vs. Unlicensed assistive personnel (UAPs) only)’ and ‘skill mix on the day shift (compared with night shift)’.

### 3.4 Nursing‐sensitive patient outcomes

Twenty‐six outcomes sensitive to nursing care were identified in the reviewed studies. These indicators were: length of stay, gastric ulcer/gastritis/upper gastrointestinal bleeding, acute myocardial infarction, restraint use, failure‐to‐rescue, pneumonia, sepsis, urinary tract infections, mortality, pressure injury, infections (excluding urinary tract infections), shock/cardiac arrest/heart failure, falls and falls injury, deep vein thrombosis, central nervous system complications, pulmonary failure or pulmonary embolism, medication error, physiological/metabolic derangement, pain control, ventilator weaning, patient wait time/leaving without being seen, quality of care, 30‐day readmission, postoperative respiratory failure, unplanned endotracheal tube extubation, and hypoglycaemia.

Infections (including wound infections, central line‐associated bloodstream infections, respiratory tract infections and mediastinitis) were grouped together for the purpose of data synthesis, with the exception of urinary tract infections, which were examined separately as they can account for over 30% of healthcare‐associated infections (Gardner, Mitchell, Beckingham, & Fasugba, 2014). Mortality and 30‐day mortality were also grouped together as they were often assessed simultaneously. In addition, ‘care processes relating to acute myocardial infarction’ were grouped with ‘acute myocardial infarction’ and ‘heart failure’ and ‘heart failure measures associated with care’ with ‘shock & cardiac arrest’.

### 3.5 Identified associations

Due to the heterogeneity with regard to significance and directionality of findings, patient outcomes were considered sensitive to nurse skill mix if they fulfilled the following criteria: of those studies with significant findings, three quarters or more of the studies for each patient outcome found an inverse significant relationship, that is, higher skill mix was associated with fewer adverse patient outcomes (Table 3). Non‐significant findings were also reported.

**Table 3:** Relationship between nursing skill mix and patient outcomes

Patient outcome	Number of studies	Studies reporting non‐significant outcome	Number of studies with non‐significant outcome	Significant outcome nurse skill mix	Number of studies with significant outcome	Number of significant studies where higher skill mix associated with decrease in adverse outcomes
Length of stay	13	Barkell et al. (2002), Blegen et al. (2011), Goode et al. (2011), Martsolf et al. (2014), Twigg et al. (2012), Yang et al. (2015)	6	de Cordova et al. (2014), Esparza et al. (2012), Frith et al. (2010), Kim, Park, et al. (2016), McCloskey and Diers (2005), Needleman et al. (2002), Pitkäaho et al. (2015)	7	7
Gastric ulcer/gastritis/upper gastrointestinal bleeding	6	McCloskey and Diers (2005)	1	Duffield et al. (2011), Kim and Bae (2018), Needleman et al. (2002), Roche et al. (2012), Twigg et al. (2012)	5	5
Acute myocardial infarction	4	Schreuders et al. (2015), Sochalski et al. (2008)	2	Johansen et al. (2015), Person et al. (2004)	2	2
Restraint use	2	Nil	0	Aydin et al. (2015), Staggs et al. (2016)	2	2
Failure‐to‐rescue	12	Glance et al. (2012), Schreuders et al. (2015), Sochalski et al. (2008)	3	Blegen et al. (2011), Goode et al. (2011), Needleman et al. (2002), Park et al. (2012), Roche et al. (2012), Seago et al. (2006), Twigg et al. (2016), Twigg et al. (2012), Unruh and Zang (2012)	9	8
Pneumonia	13	Barkell et al. (2002), Duffield et al. (2011), Martsolf et al. (2014), McCloskey and Diers (2005), Schreuders et al. (2015), Twigg et al. (2016)	6	Cho et al. (2003), Kim and Bae (2018), McGillis Hall et al. (2003), Needleman et al. (2002), Roche et al. (2012), Twigg et al. (2012), Unruh (2003)	7	6
Sepsis	12	Cho et al. (2003), Kim and Bae (2018), Martsolf et al. (2014), Needleman et al. (2002), Twigg et al. (2016), Twigg et al. (2012)	6	Blegen et al. (2011), Duffield et al. (2011), Goode et al. (2011), McCloskey and Diers (2005), Roche et al. (2012), Unruh and Zang (2012)	6	5
Urinary tract infections	18	Barkell et al. (2002), Cho et al. (2003), Duffield et al. (2011), Martsolf et al. (2014), McGillis Hall and Doran (2004), McGillis Hall et al. (2004), Sovie and Jawad (2001), Yang (2003)	7	Esparza et al. (2012), Frith et al. (2010), Kim and Bae (2018), McCloskey and Diers (2005), Needleman et al. (2002), Roche et al. (2012), Schreuders et al. (2015), Twigg et al. (2016), Twigg et al. (2012), Unruh (2003), Yang et al. (2015)	11	9
Mortality/30‐day mortality	17	Blegen et al. (2011), Martsolf et al. (2014), Needleman et al. (2002), Schreuders et al. (2015), Sochalski et al. (2008), Twigg et al. (2012)	6	Aiken et al. (2016), Ambrosi et al. (2017), Ball et al. (2018), Estabrooks et al. (2005), Glance et al. (2012), Goode et al. (2011), He et al. (2013), Kim and Bae (2018), McCloskey and Diers (2005), Tourangeau et al. (2002), Twigg et al. (2016)	11	9
Pressure injury	24	Bae et al. (2014), Blegen et al. (2011), Bolton et al. (2007), Cho et al. (2003), Donaldson et al. (2005), Duffield et al. (2011), Goode et al. (2011), Needleman et al. (2002), Schreuders et al. (2015), Sovie and Jawad (2001), Unruh and Zang (2012), Yang et al. (2015), Yang (2003), Twigg et al. (2016)	14	Boyle et al. (2016), Choi and Staggs (2014), Frith et al. (2010), He et al. (2016), Leary et al. (2016), McCloskey and Diers (2005), Roche et al. (2012), Schneider et al.(2016), Twigg et al. (2012), Unruh (2003)	10	8
Infections [excluding urinary tract infections]	15	Cho et al. (2003), Duffield et al. (2011), Goode et al. (2011), Martsolf et al. (2014), Twigg et al. (2012), Unruh (2003), Yang (2003)	7	Blegen et al. (2011), Frith et al. (2010), Kim and Bae (2018), McCloskey and Diers (2005), McGillis Hall and Doran (2004), McGillis Hall et al. (2004), Schreuders et al. (2015), Unruh and Zang (2012), Yang et al. (2015)	8	6
Shock/cardiac arrest/Heart failure	8	Blegen et al. (2011), McCloskey and Diers (2005), Newhouse et al. (2013), Schreuders et al. (2015)	4	Duffield et al. (2011), Kim and Bae (2018), Needleman et al. (2002), Twigg et al. (2012)	4	3
Falls & injury falls	18	Breckenridge‐Sproat et al. (2012), Donaldson et al. (2005), Leary et al. (2016) Lee et al. (2005), McGillis Hall and Doran (2004), McGillis Hall et al. (2004), Potter et al. (2003), Yang (2003)	7	Aydin et al. (2015), Bae et al. (2014), Bolton et al. (2007), Boyle et al. (2016), He et al. (2016), Patrician et al. (2011), Staggs and Dunton (2014), Staggs et al. (2012), Twigg et al. (2016), Tzeng et al. (2011), Unruh (2003)	11	6
Deep vein thrombosis	7	Duffield et al. (2011), Kim and Bae (2018), Martsolf et al. (2014), Needleman et al. (2002), Schreuders et al. (2015)	5	McCloskey and Diers (2005), Twigg et al. (2012)	2	1
Central nervous system complications	6	Duffield et al. (2011), Kim and Bae (2018), Needleman et al. (2002), Twigg et al. (2012)	4	McCloskey and Diers (2005), Roche et al. (2012)	2	1
Pulmonary failure/pulmonary embolism	5	Kim and Bae (2018), Martsolf et al. (2014), Twigg et al. (2012)	3	Duffield et al. (2011), McCloskey and Diers (2005)	2	1
Medication error	5	Chang and Mark (2011), Lee et al. (2005), Potter et al. (2003)	3	McGillis Hall and Doran (2004), McGillis Hall et al. (2004), Yang et al. (2015)	2	1
Physiological/metabolic derangement	5	Kim and Bae (2018), Needleman et al. (2002), Twigg et al. (2012)	3	Duffield et al. (2011), McCloskey and Diers (2005)	2	1
Pain control	2	Nil	0	Huston (2001), McGillis Hall et al. (2003)	2	1
Ventilator weaning	1	Nil	0	Yang et al. (2015)	1	1
Patient wait time	1	Nil	0	Paulson (2004)	1	1
Quality of care	1	Nil	0	McGillis Hall and Doran (2004)	1	1
30 day readmission	1	Nil	0	Kim and Bae (2018)	1	1
Post‐operative respiratory failure	1	Martsolf et al. (2014)	1	Nil	0	Nil
Hypoglycaemia	1	Nil	0	Anthony (2008)	1	Nil
Unplanned endotracheal tube extubation	1	Yang et al. (2015)	1	Nil	0	Nil

Based on these guidelines, 12 outcomes met the criteria for a higher skill mix associated with a decrease in adverse outcomes. These outcomes were: length of stay, ulcer, gastritis and upper gastrointestinal bleeds, acute myocardial infarction, restraint use, failure‐to‐rescue, pneumonia, sepsis, urinary tract infection, mortality/30‐day mortality, pressure injury, infections (excluding urinary tract infections) and shock/cardiac arrest/heart failure.

Thirteen studies examined skill mix and length of stay, six of which did not report a statistically significant relationship. Of the seven reporting significant results, all found a higher proportion of skill mix was associated with lower length of stay. Six studies examined skill mix and gastritis & upper gastrointestinal bleeds of which one did not report significant results. Of the five significant results, all found an increase in skill mix resulted is a decrease in gastritis & upper gastrointestinal bleeds. Four studies examined skill mix and acute myocardial infarction, two with significant results both of which found an increase in skill mix was associated with a decrease in acute myocardial infarction. Two studies did not have any significant findings. Two studies examined restraint use and both were significant finding a decrease in restraint use with an increase in skill mix (see Table 3).

A further eight patient outcomes were found to have decreased adverse outcomes with a higher skill mix in three quarters or more of the studies with significant findings reviewed for each outcome. Twelve studies examined failure to rescue of which three did not report a statistically significant relationship. Of the nine significant findings, eight of these identified an inverse relationship between higher skill mix and fewer failure to rescue events. Thirteen studies examined pneumonia, six of which did not report a statistically significant relationship. Seven studies had significant findings about pneumonia, with six inversely related to skill mix. Twelve studies examined sepsis and six studies did not report a significant relationship between skill mix and sepsis. Six studies did report significant findings about sepsis and five were inversely related to skill mix. Eighteen studies examined urinary tract infections of which seven did not report significant results. Eleven did report significant findings about urinary tract infections and nine of these were inversely related to skill mix. Seventeen studies examined mortality and six did not report significant findings. Eleven studies had significant findings about mortality and nine of these were inversely related to skill mix. Twenty‐four studies examined pressure injuries, of which fourteen did not report significant findings. Of the 10 studies reporting significant findings, most (*N* = 8) reported that a higher skill mix was associated with fewer pressure injuries. Fifteen studies examined infections (excluding urinary tract infections) of which seven did not report significant findings. Of the eight studies with significant findings, six were inversely related to skill mix. Finally, eights studies examined shock/cardiac arrest/heart failure of which four did not report significant findings. Of the four significant findings, three of these were inversely related to skill mix (see Table 3). However, one study examining heart failure (Newhouse et al., 2013) was undertaken using a RTC and found a non‐significant outcome for the effect of skill mix.

The remainder of the patient outcomes examined were inconclusive. Eighteen studies examined the relationship between skill mix and falls and falls injury, seven did not report a significant relationship. Of the eleven studies with significant results, six studies found an inverse relationship and five did not, providing mixed results. Deep vein thrombosis, central nervous system complications, pulmonary failure/pulmonary embolism, medication error, physiologic/metabolic derangement and pain control had fewer studies (2–7) per outcome and in each occasion only two studies were significant. Of these, again in each case, one of the two had an inverse relationship with skill mix, again providing mixed results (see Table 3). In addition, five patient outcomes were excluded as although they technically satisfied the stated inclusion criteria, they were not considered suitable outcomes as only one study respectively investigated these outcomes. These were ventilator weaning, hypoglycaemia, patient wait time, quality of care, and 30‐day readmission (Table 3). One patient outcome, blood incompatibilities, was not included in the final synthesis, as the study in question did not find any occurrences of the outcome during their data collection (Frith et al., 2010).

Table 3 outlines the studies that found a significant relationship between nursing skill mix and those that did not, as well as the proportion of significant studies according to directionality, that is, whether a higher skill mix containing more RNs was associated with an increase or decrease in adverse patient outcomes.

## 4 DISCUSSION

### 4.1 Summary of evidence

Out of 26 patient outcomes reported in the 63 studies included in this review, 12 showed an inverse association with skill mix, when assessed using pre‐defined criteria. These outcomes were: length of stay, ulcer, gastritis and upper gastrointestinal bleeds, acute myocardial infarction, restraint use, failure‐to‐rescue, pneumonia, sepsis, urinary tract infection, mortality/30‐day mortality, pressure injury, infections (excluding urinary tract infections) and shock/cardiac arrest/heart failure. There was a reduction in these 12 patient outcomes when a higher nursing skill mix containing more Registered Nurses was present. These results are similar to relationships found in regard to a systematic review of nurse staffing hours (Kane et al., 2007) where mortality, hospital acquired pneumonia, unplanned extubation, cardiac arrest in ICUs, risk of failure to rescue in surgical patients and length of stay was shorter in ICUs and in surgical patients. Additionally, a systematic review of acute specialist units found higher staffing levels were associated with reduced mortality, medication errors, ulcers, restraint use, infections, and pneumonia (Driscoll et al., 2017).

Any relationship between the remainder of the patient outcomes and skill mix was inconclusive. These outcomes were falls and falls injury, deep vein thrombosis, central nervous system complications, pulmonary failure/pulmonary embolism, medication error (in contrast to Driscoll et al. (2017)), physiologic/metabolic derangement, and pain control. Five patient outcomes were excluded as not being suitable as only one study investigated each outcome. These were ventilator weaning, hypoglycaemia, patient wait time, quality of care and 30‐day readmission.

Since the seminal study conducted in the 1980’s found that interaction and coordination amongst clinicians (medical and nursing staff) reduced patient deaths in ICU settings, nurse staffing has been under the research microscope (Knaus, Draper, Wagner, & Zimmerman, 1986). Fundamental to this research is the recognition that Registered Nurses provide a continuous (24 hr per day, 7 days per week) surveillance system for patients. This surveillance system enables early detection and prompt intervention when a patient's condition deteriorates, or health issues emerge (Twigg, Duffield, Thompson, & Rapley, 2010). The ability of nurses to undertake this critical role is dependent on the hours of care and the skill mix of those providing that care. It is not surprising, therefore, that this review found 12 outcomes inversely related to skill mix. What remains less well understood is how nurses manage less than desired hours of care or skill mix. Emerging research suggests nurses ration their care when there is insufficient nurses or lower skill mix and prioritize the most urgent or critical aspects of care and some care may be altogether missed (Bail & Grealish, 2016). How this might have an impact on patient outcomes over their length of stay is less well understood but this care rationing may provide some explanation as to why skill mix is associated with only some but not all patient outcomes (Griffiths et al., 2018).

Many decades that have now passed since the first seminal works in this area, however, the research examining the association between skill mix and patient outcomes remains in an exploratory stage evidenced by the number of outcomes studied and the number of non‐significant results. The research is plagued by methodological issues including; many research studies rely on secondary administrative data to recode patient discharge diagnoses into adverse events, different measures and definitions are still being used for staffing and skill mix variables as well as patient outcomes reducing the comparability of results and data and coding processes often vary or are not transparent (Unruh & Zhang, 2012).

### 4.2 Limitations

The results of this quantitative systematic review should be interpreted with caution. The methodological quality of the included studies is far from ideal, with only very few studies using experimental designs. In this review, only three out of the sixty‐three studies included in the narrative summary employed experimental or quasi‐experimental designs. Many of the studies collected self‐reported data using single measurement methods, for example, self‐reported pain scales (Huston, 2001) and self‐reported use of falls risk assessment and policy use (Aydin, Donaldson, Aronow, Fridman, & Brown, 2015), raising the potential for common method bias (Podsakoff, MacKenzie, & Podsakoff, 2011; Wingate, Sng, & Loprinzi, 2018). Self‐reported studies may inflate, deflate or have no effect on the relationships being studied due to common method bias (Podsakoff et al., 2011). Hence, these results may have over‐ or underestimated the effect of skill mix on patient outcomes. Whilst common method bias has been identified as an issue for self‐reported studies, the studies did not discuss how common method bias was addressed. Common method bias can be decreased by the use of different data collection methods, times and locations and checking the wording, number of items and placement of items in data collection tools to maximize motivation and minimize task difficulty to encourage participants to provide accurate results (Podsakoff et al., 2011; Wingate et al., 2018).

There was also a large degree of variety of heterogeneity in the definitions and of research methods used for determining the effect of skill mix on patient outcomes makes comparison of data difficult. This review was only able to provide a narrative analysis of results as meta‐analysis could not be performed. Consequently, inferences about causal relationships between nurse skill mix and nursing‐sensitive patient outcomes cannot be drawn.

## 5 CONCLUSION

Of those studies identifying significant results, there were 12 outcomes where three quarters or more of those studies found an inverse association with increases in nurse skill mix: length of stay, ulcer, gastritis and upper gastrointestinal bleeds, acute myocardial infarction, restraint use, failure‐to‐rescue, pneumonia, sepsis, urinary tract infection, mortality/30‐day mortality, pressure injury, infections (excluding urinary tract infections), and shock/cardiac arrest/heart failure. Nevertheless, there was insufficient evidence to draw inferences about causal relationships since research into nursing‐sensitive outcomes continues to suffer from methodological flaws and heterogeneity of results. This has an impact on the synthesis of research findings and recommendations for future research and policy. More experimental or longitudinal study designs are required, which have the potential to establish causal relationships. Currently, a plethora of studies investigating interactions between nurse skill mix and patient outcomes employ observational designs that lack control of basic confounding variables.

Furthermore, future research must not only produce a widely agreed definition of nurse skill mix and its most appropriate form of measurement but must also investigate the existence of an optimal level of RNs in skill mix and explore potential non‐linear relationships between nursing skill mix and patient outcomes. Nonetheless, this systematic review suggests that critical patient outcomes such as mortality, failure‐to‐rescue, and length of stay can be improved with a higher skill mix. Those making staffing decisions cannot ignore this association.

## CONFLICT OF INTEREST

No conflict of interest has been declared by the authors.
