# Supplementary material for: A quantitative systematic review of the association between nurse skill mix and nursing‐sensitive patient outcomes in the acute care setting
Source: J Adv Nurs. 2019 Oct 3;75(12):3404–23. doi: 10.1111/jan.14194 (PMC6899638; doi:10.1111/jan.14194)
Supplement: Supplementary file 1 [file JAN-75-3404-s001.pdf]

Online supplement A: Systematic Review Protocol

Measuring the effect of nurse skill mix upon patient outcomes (nurse sensitive outcomes) in the acute care setting

Research questions

What is the effect of changing the nursing skill mix on patient outcomes (sensitive to nursing care) in adult patients in acute care hospitals?

PICOS (Population, Intervention, Comparison, Intervention, Study Design) statement

Population: adult patients in acute care hospital wards.
Intervention: a particular nursing skill mix level.
Comparator: a different nursing skill mix level.
Outcome: patient outcomes (sensitive to nursing care) such as mortality, deep vein thrombosis, sepsis, urinary tract infection, pressure injuries, pneumonia, upper gastro-intestinal bleeding, shock/cardiac arrest, central nervous system complications, surgical wound infections, pulmonary failure, physiologic/metabolic derangement or any others identified (and tested) by researchers as outcomes potentially sensitive to nursing care.
Study design: Observational/Descriptive (includes cross-sectional, prospective/cohort studies, case-control studies) and Experimental (includes experimental (RCT) and quasi-experimental (time-series etc))

Databases

Databases to be searched include Cochrane Library, CINAHL Plus with Full Text, MEDLINE, Scopus, Web of Science and Joanna Briggs Institute with article inclusion from 2000 to the present.

Search terms

The search terms will cover two main concepts.
1. Nurse skill mix
2. Patient outcomes

Skill mix refers to the mix of nursing staff, both registered and unregistered, on a ward. Dependent on country, the nurse mix may include registered nurses, enrolled nurses, licenced practical nurses, certified nursing assistants, assistants in nursing, healthcare assistants …

Search strategy

(RN mix OR skill mix OR registered nurses OR nursing assistants OR practical nurses)
AND
(Nurs* sensitive outcome* OR mortality OR sepsis OR urinary tract infection OR pressure ulcer OR pressure injury OR pneumonia OR failure to rescue OR clinical deterioration OR Outcomes (Health Care) OR Outcome Assessment OR Quality of Health Care OR Nursing Outcomes OR Outcomes Research OR Patient Safety OR Health Care Errors OR Accidental Falls OR Surgical Wound Infection OR Venous Thrombosis OR Shock OR Heart Arrest OR Medication Errors OR Infection OR Respiratory Failure OR Gastrointestinal Hemorrhage OR Central Nervous System Infections OR Metabolic Diseases)

Inclusion criteria

Articles written in the English language
Articles including adult patients in acute care settings.

Exclusion criteria

Articles in a language other than English
Articles including patients in peri-operative settings, maternity patients, paediatric (age<18 years) patients, newborns, mental health and substance abuse settings; and palliative care patients.
