# Supplementary material for: A quantitative systematic review of the association between nurse skill mix and nursing‐sensitive patient outcomes in the acute care setting
Source: J Adv Nurs. 2019 Oct 3;75(12):3404–23. doi: 10.1111/jan.14194 (PMC6899638; doi:10.1111/jan.14194)
Supplement: Supplementary file 2 [file JAN-75-3404-s002.pdf]

Online supplement B: Electronic database search strategy (1st January 2000 to 30th September 2018)

CINAHL Plus with Full Text (EEBSCO Host)
((((nurs* sensitive outcome OR mortality OR sepsis OR urinary tract infection OR pressure ulcer OR pressure injury OR pneumonia OR failure to rescue OR clinical deterioration OR outcomes health care OR outcome assessment OR quality of health care) OR ((nursing outcomes) OR (outcomes research) OR (patient safety) OR (health care errors) OR (accidental falls) OR (surgical wound infection) OR (venous thrombosis) OR (failure to rescue) OR (shock) OR (heart arrest) OR (medication errors) OR (infection)) OR ((respiratory failure) OR (gastrointestinal hemorrhage) OR (central nervous system infections) OR (metabolic disease)))) AND (((RN mix) OR (Skill mix) OR (registered nurse) OR (nursing assistant) OR (practical nurse)))) NOT (((nursing home) OR (neonatal) OR (maternity) OR (paediatric) OR (mental health) OR (psych*) OR (children) OR (midwifery) OR (intensive care) OR (palliative care) OR (critical care)))
Limiters - Published Date: 20000101-20180931; Human; Language: English
Expanders - Apply related words
Search modes - Boolean/Phrase

MEDLINE
((((nurs* sensitive outcome OR mortality OR sepsis OR urinary tract infection OR pressure ulcer OR pressure injury OR pneumonia OR failure to rescue OR clinical deterioration OR outcomes health care OR outcome assessment OR quality of health care) OR ((nursing outcomes) OR (outcomes research) OR (patient safety) OR (health care errors) OR (accidental falls) OR (surgical wound infection) OR (venous thrombosis) OR (failure to rescue) OR (shock) OR (heart arrest) OR (medication errors) OR (infection)) OR ((respiratory failure) OR (gastrointestinal hemorrhage) OR (central nervous system infections) OR (metabolic disease)))) AND (((RN mix) OR (Skill mix) OR (registered nurse) OR (nursing assistant) OR (practical nurse)))) NOT (((nursing home) OR (neonatal) OR (maternity) OR (paediatric) OR (mental health) OR (psych*) OR (children) OR (midwifery) OR (intensive care) OR (palliative care) OR (critical care)))
Limiters - Date of Publication: 20000101-20181231; English Language; Language: English
Expanders - Apply related words
Search modes - Boolean/Phrase
MeSH terms - nursing assistants; mortality; sepsis; urinary tract infection; pneumonia; clinical deterioration; quality of health care; patient safety; accidental falls; surgical wound infection; venous thrombosis; shock; heart arrest; medication errors; infection; central nervous system infections

Cochrane Library (EEBSCO Host)
((((nurs* sensitive outcome OR mortality OR sepsis OR urinary tract infection OR pressure ulcer OR pressure injury OR pneumonia OR failure to rescue OR clinical deterioration OR outcomes health care OR outcome assessment OR quality of health care) OR ((nursing outcomes) OR (outcomes research) OR (patient safety) OR (health care errors) OR (accidental falls) OR (surgical wound infection) OR (venous thrombosis) OR (failure to rescue) OR (shock) OR (heart arrest) OR (medication errors) OR (infection)) OR ((respiratory failure) OR (gastrointestinal hemorrhage) OR (central nervous system infections) OR (metabolic disease)))) AND (((RN mix) OR (Skill mix) OR (registered nurse) OR (nursing assistant) OR (practical nurse)))) NOT (((nursing home) OR (neonatal) OR (maternity) OR (paediatric) OR (mental health) OR (psych*) OR (children) OR (midwifery) OR (intensive care) OR (palliative care) OR (critical care))) in Title Abstract Keyword - (Word variations have been searched)

Web of science
(TS=(((((nurs* sensitive outcome OR mortality OR sepsis OR urinary tract infection OR pressure ulcer OR pressure injury OR pneumonia OR failure to rescue OR clinical deterioration OR outcomes health care OR outcome assessment OR quality of health care) OR ((nursing outcomes) OR (outcomes research) OR (patient safety) OR (health care errors) OR (accidental falls) OR (surgical wound infection) OR (venous thrombosis) OR (failure to rescue) OR (shock) OR (heart arrest) OR (medication errors) OR (infection)) OR ((respiratory failure) OR (gastrointestinal hemorrhage) OR (central nervous system infections) OR (metabolic disease)))) AND (((RN mix) OR (Skill mix) OR (registered nurse) OR (nursing assistant) OR (practical nurse)))) NOT (((nursing home) OR (neonatal) OR (maternity) OR (paediatric) OR (mental health) OR (psych*) OR (children) OR (midwifery) OR (intensive care) OR (palliative care) OR (critical care))))) *AND* **LANGUAGE:** (English) Indexes=SCI-EXPANDED, SSCI, A&HCI, CPCI-S, CPCI-SSH, ESCI, CCR-EXPANDED, IC Timespan=2000-2018

Scopus
(ALL ("rn mix" OR "skill mix" OR "registered nurses" OR "nursing assistants" OR "practical nurses")) AND (ALL ("nurs* sensitive outcome" OR mortality OR sepsis OR "urinary tract infection" OR "pressure ulcer" OR "pressure injury" OR pneumonia OR "failure to rescue" OR "clinical deterioration" OR "outcomes health care" OR "outcome assessment" OR "quality of health care" OR "nursing outcomes" OR "outcomes research" OR "patient safety" OR "health care errors" OR "accidental falls" OR "surgical wound infection " OR "venous thrombosis" OR shock OR "heart arrest" OR "medication errors" OR infection OR "respiratory failure" OR "gastrointestinal hemorrhage" OR "central nervous system infections" OR "metabolic disease")) AND NOT ("nursing home" OR neonatal OR maternity OR paediatric OR pediatric OR "mental health" OR psych* OR children OR midwifery OR "intensive care" OR "palliative care" OR "critical care") AND (LIMIT-TO (LANGUAGE , "English")) AND (LIMIT-TO (PUBYEAR , 2018) OR LIMIT-TO (PUBYEAR , 2017) OR LIMIT-TO (PUBYEAR , 2016) OR LIMIT-TO (PUBYEAR , 2015) OR LIMIT-TO (PUBYEAR , 2014) OR LIMIT-TO (PUBYEAR , 2013) OR LIMIT-TO (PUBYEAR , 2012) OR LIMIT-TO (PUBYEAR , 2011) OR LIMIT-TO (PUBYEAR , 2010) OR LIMIT-TO (PUBYEAR , 2009) OR LIMIT-TO (PUBYEAR , 2008) OR LIMIT-TO (PUBYEAR , 2007) OR LIMIT-TO (PUBYEAR , 2006) OR LIMIT-TO (PUBYEAR , 2005) OR LIMIT-TO (PUBYEAR , 2004) OR LIMIT-TO (PUBYEAR , 2003) OR LIMIT-TO (PUBYEAR , 2002) OR LIMIT-TO (PUBYEAR , 2001) OR LIMIT-TO (PUBYEAR , 2000))

JBI (OVID)
(((nurs* sensitive outcome or mortality or sepsis or urinary tract infection or pressure ulcer or pressure injury or pneumonia or failure to rescue or clinical deterioration or outcomes health care or outcome assessment or quality of health care or (nursing outcomes or outcomes research or patient safety or health care errors or accidental falls or surgical wound infection or venous thrombosis or failure to rescue or shock or heart arrest or medication errors or infection) or (respiratory failure or gastrointestinal hemorrhage or central nervous system infections or metabolic disease)) and (RN mix or Skill mix or registered nurse or nursing assistant or practical nurse)) not (nursing home or neonatal or maternity or paediatric or mental health or psych* or children or midwifery or intensive care or palliative care or critical care)).mp. [mp=text, heading word, subject area node, title]
limit 1 to yr="2000 - 2018"

Embase strategy

('nurse sensitive outcome' OR (('nurse'/exp OR nurse) AND sensitive AND ('outcome'/exp OR outcome)) OR 'mortality'/exp OR mortality OR 'sepsis'/exp OR sepsis OR 'urinary tract infection'/exp OR 'urinary tract infection' OR (urinary AND ('tract'/exp OR tract) AND ('infection'/exp OR infection)) OR 'pressure ulcer'/exp OR 'pressure ulcer' OR (('pressure'/exp OR pressure) AND ('ulcer'/exp OR ulcer)) OR 'pressure injury'/exp OR 'pressure injury' OR (('pressure'/exp OR pressure) AND ('injury'/exp OR injury)) OR 'pneumonia'/exp OR pneumonia OR 'clinical deterioration'/exp OR 'clinical deterioration' OR (('clinical'/exp OR clinical) AND ('deterioration'/exp OR deterioration)) OR 'outcomes health care' OR (('outcomes'/exp OR outcomes) AND ('health'/exp OR health) AND ('care'/exp OR care)) OR 'outcome assessment'/exp OR 'outcome assessment' OR (('outcome'/exp OR outcome) AND ('assessment'/exp OR assessment)) OR 'quality of health care'/exp OR 'quality of health care' OR (('quality'/exp OR quality) AND of AND ('health'/exp OR health) AND ('care'/exp OR care)) OR 'nursing outcomes' OR (('nursing'/exp OR nursing) AND ('outcomes'/exp OR outcomes)) OR 'outcomes research'/exp OR 'outcomes research' OR (('outcomes'/exp OR outcomes) AND ('research'/exp OR research)) OR 'patient safety'/exp OR 'patient safety' OR (('patient'/exp OR patient) AND ('safety'/exp OR safety)) OR 'health care errors' OR (('health'/exp OR health) AND ('care'/exp OR care) AND errors) OR 'accidental falls'/exp OR 'accidental falls' OR (accidental AND ('falls'/exp OR falls)) OR 'surgical wound infection'/exp OR 'surgical wound infection' OR (surgical AND ('wound'/exp OR wound) AND ('infection'/exp OR infection)) OR 'venous thrombosis'/exp OR 'venous thrombosis' OR (venous AND ('thrombosis'/exp OR thrombosis)) OR 'failure to rescue'/exp OR 'failure to rescue' OR (('failure'/exp OR failure) AND to AND ('rescue'/exp OR rescue)) OR 'shock'/exp OR shock OR 'heart arrest'/exp OR 'heart arrest' OR (('heart'/exp OR heart) AND ('arrest'/exp OR arrest)) OR 'medication errors'/exp OR 'medication errors' OR (('medication'/exp OR medication) AND errors) OR 'infection'/exp OR infection OR 'respiratory failure'/exp OR 'respiratory failure' OR (respiratory AND ('failure'/exp OR failure)) OR 'gastrointestinal hemorrhage'/exp OR 'gastrointestinal hemorrhage' OR (gastrointestinal AND ('hemorrhage'/exp OR hemorrhage)) OR 'central nervous system infections'/exp OR 'central nervous system infections' OR (('central'/exp OR central) AND nervous AND system AND ('infections'/exp OR infections)) OR 'metabolic disease'/exp OR 'metabolic disease' OR (metabolic AND ('disease'/exp OR disease))) AND ('rn mix' OR (rn AND mix) OR 'skill mix'/exp OR 'skill mix' OR (('skill'/exp OR skill) AND mix) OR 'registered nurse'/exp OR 'registered nurse' OR (registered AND ('nurse'/exp OR nurse)) OR 'nursing assistant'/exp OR 'nursing assistant' OR (('nursing'/exp OR nursing) AND ('assistant'/exp OR assistant)) OR 'practical nurse'/exp OR 'practical nurse' OR (practical AND ('nurse'/exp OR nurse))) NOT ('nursing home'/exp OR 'nursing home' OR (('nursing'/exp OR nursing) AND ('home'/exp OR home)) OR neonatal OR 'maternity'/exp OR maternity OR 'paediatric'/exp OR paediatric OR 'mental health'/exp OR 'mental health' OR (mental AND ('health'/exp OR health)) OR psych* OR 'children'/exp OR children OR 'midwifery'/exp OR midwifery OR 'intensive care'/exp OR 'intensive care' OR (intensive AND ('care'/exp OR care)) OR 'palliative care'/exp OR 'palliative care' OR (palliative AND ('care'/exp OR care)) OR 'critical care'/exp OR 'critical care' OR (critical AND ('care'/exp OR care))) AND [2000-2018]/py
