# Supplementary material for: A quantitative systematic review of the association between nurse skill mix and nursing‐sensitive patient outcomes in the acute care setting
Source: J Adv Nurs. 2019 Oct 3;75(12):3404–23. doi: 10.1111/jan.14194 (PMC6899638; doi:10.1111/jan.14194)
Supplement: Supplementary file 3 [file JAN-75-3404-s003.pdf]

Online supplement C1. Critical Appraisal results for included studies using the JBI - Analytical Cross Sectional Study 2017 Checklist

Source	1	2	3	4	5	6	7	8	Decision
Ball et al. (2018)	U	N	Y	Y	Y	Y	Y	Y	Include*
Boyle et al. (2016)	Y	Y	Y	Y	Y	Y	Y	Y	Include*
Kim and Bae (2018)	Y	Y	Y	Y	U	U	Y	Y	Include*
Kim, Park et al. (2016)	Y	N	Y	Y	Y	NA	Y	Y	Include*
Leary, A., et al. (2016)	Y	Y	Y	Y	N	N	Y	Y	Include*
Irvin et al. (2017)	-	-	-	-	-	-	-	-	Exclude§
Kim and Han (2018)	-	-	-	-	-	-	-	-	Exclude§
Roche et al. (2016)	-	-	-	-	-	-	-	-	Exclude§
Sharma et al. (2016)	-	-	-	-	-	-	-	-	Exclude§
Unruh and Hofler (2016)	-	-	-	-	-	-	-	-	Exclude§

Y – Yes, N- No, U- Unclear

Analytical Cross Sectional Study 2017 (8 questions): 1) Were the criteria for inclusion in the sample clearly defined? 2) Were the study subjects and the setting described in detail? 3) Was the exposure measured in a valid and reliable way? 4) Were objective, standard criteria used for measurement of the condition? 5) Were confounding factors identified? 6) Were strategies to deal with confounding factors stated? 7) Were the outcomes measured in a valid and reliable way? 8) Was appropriate statistical analysis used?

*Reason for inclusion:-Reported nurse sensitive outcome measures using statistical analysis

§ Reasons for exclusion: -Not skill mix; not acute; not nurse sensitive outcome

Online supplement C2. Critical Appraisal results for included studies using the JBI – Cohort/Case Control 2015 Checklist

Source	1	2	3	4	5	6	7	8	9	Decision
Blegen et al. (2011)	N	N	Y	Y	Y	Y	U	Y	Y	Include*
Bolton et al. (2007)	Y	U	Y	Y	Y	Y	U	Y	Y	Include*
Breckenridge-Sproat et al. (2012)	N	U	U	Y	Y	Y	U	Y	Y	Include*
de Cordova et al. (2014)	N	U	Y	Y	Y	Y	N	Y	Y	Include*
Donaldson et al. (2005)	Y	Y	U	U	Y	Y	N	Y	Y	Include*
Duffield et al. (2011)	Y	U	Y	Y	Y	Y	N	Y	Y	Include*
Esparza et al. (2012)	Y	Y	Y	Y	Y	U	U	Y	Y	Include*
Estabrooks et al. (2005)	Y	U	Y	Y	Y	U	N	Y	Y	Include*
Frith et al. (2010)	Y	Y	Y	Y	Y	Y	N	Y	Y	Include*
Glance et al. (2012)	U	U	Y	Y	Y	U	U	Y	Y	Include*
Martsolf et al. (2014)	U	U	U	Y	Y	Y	U	Y	Y	Include*
McGillis Hall, & Doran (2004)	U	N	N	Y	N	U	U	Y	Y	Include*
McGillis Hall, Doran & Pink (2004)	N	N	Y	Y	Y	U	U	Y	Y	Include*
McGillis Hall, Doran, Baker et al. (2003)	Y	U	U	Y	Y	Y	Y	Y	Y	Include*
Sovie & Jawad (2001)	Y	Y	U	Y	Y	Y	U	Y	Y	Include*
Staggs & Dunton (2014)	N	U	U	Y	Y	U	U	Y	Y	Include*
Staggs et al. (2012)	N	N	N	Y	Y	Y	U	Y	Y	Include*
Tourangeau et al. (2002)	Y	Y	Y	Y	Y	Y	Y	Y	Y	Include*
Twigg, D. et al. (2012)	N	Y	U	Y	Y	Y	U	Y	Y	Include*
Unruh (2003)	U	N	N	Y	Y	Y	U	Y	Y	Include*
Unruh and Zang (2012)	Y	N	U	Y	Y	Y	U	Y	Y	Include*
Yang et al. (2015)	Y	Y	U	Y	Y	Y	Y	Y	Y	Include*
Furukawa et al. (2011)	Y	U	Y	Y	Y	Y	U	Y	Y	Exclude§
Harless, D. W. and B. A. Mark (2010)	-	-	-	-	-	-	-	-	-	Exclude§
Mark et al. (2004)	U	y	y	y	y	y	U	Y	Y	Exclude§
Rochefort et al. (2016)	-	-	-	-	-	-	-	-		Exclude§
Thomas-Hawkins, C. et al. (2008)	N	U	U	Y	U	Y	Y	Y	Y	Exclude§
Tourangeau (2002)	-	-	-	-	-	-	-	-	-	Exclude§
Tschannen & Kalisch (2009)	N	Y	U	Y	Y	N	Y	Y	Y	Exclude§
Unruh (2008)	-	-	-	-	-	-	-	-	-	Exclude§
Yang et al. (2012)	N	Y	U	Y	Y	Y	U	Y	Y	Exclude§

Y – Yes, N- No, U- Unclear
Cohort/Case Control 2015 (9 questions): 1) Is sample representative of patients in the population as a whole? 2) Are the patients at a similar point in the course of their condition/illness? 3) Has bias been minimized in relation to selection of cases and of controls? 4) Are confounding factors identified and strategies to deal with them stated? 5) Are outcomes assessed using objective criteria? 6) Was follow up carried out over a sufficient time period? 7) Were the outcomes of people who withdrew described and included in the analysis? 8) Were outcomes measured in a reliable way? 9) Was appropriate statistical analysis used?
*Reason for inclusion:-Reported nurse sensitive outcome measures using statistical analysis
§ Reasons for exclusion: -Not skill mix; nursing outcomes (not patients); conference abstract; summary of other research; review paper; unequal comparison groups

Online supplement C3. Critical Appraisal results for included studies using the JBI - Descriptive/case series studies 2015 checklist

Source	1	2	3	4	5	6	7	8	9	Decision
Aiken et al. (2016)	U	Y	y	U	U	Y	U	U	Y	Include*
Ambrosi et al. (2017)	N	Y	Y	Y	Y	Y	U	Y	U	Include*
Anthony (2008)	N	Y	N	Y	Y	Y	U	Y	N	Include*
Aydin et al. (2015)	N	N	Y	Y	Y	Y	U	Y	Y	Include*
Bae et al. (2014)	Y	Y	Y	Y	Y	U	U	Y	Y	Include*
Barkell et al. (2002)	N	Y	Y	Y	Y	U	Y	Y	Y	Include*
Chang & Mark (2011)	Y	Y	Y	Y	Y	U	N	Y	Y	Include*
Cho et al. (2003)	U	U	Y	N	Y	U	U	U	U	Include*
Choi and Staggs (2014)	U	Y	Y	Y	Y	U	U	Y	Y	Include*
Goode et al. (2011)	N	Y	Y	Y	Y	Y	N	Y	Y	Include*
He et al. (2013)	N	Y	Y	Y	Y	U	U	Y	Y	Include*
He et al. (2016)	N	Y	N	Y	U	Y	U	Y	Y	Include*
Huston, C. J. (2001)	Y	Y	N	Y	Y	Y	U	Y	Y	Include*
Johansen et al. (2015)	N	Y	Y	U	U	Y	U	U	Y	Include*
Kim, Kim et al. (2016)	N	Y	Y	Y	U	Y	U	Y	Y	Include*
Lake et al. (2010)	N	Y	Y	Y	Y	Y	U	Y	Y	Include*
McCloskey and Diers (2005)	N	Y	N	Y	U	Y	U	Y	Y	Include*
Needleman et al. (2002) N Engl J Med	N	Y	Y	Y	Y	Y	U	Y	Y	Include*
Park et al. (2012)	N	Y	Y	Y	U	Y	U	Y	Y	Include*
Patrician et al. (2011)	N	Y	Y	Y	Y	Y	U	Y	Y	Include*
Patrician et al. (2016)	N	Y	Y	Y	Y	Y	U	Y	Y	Include*
Paulson (2004)	N	Y	N	Y	Y	Y	U	Y	Y	Include*
Person et al. (2004)	Y	Y	Y	Y	Y	Y	N	Y	Y	Include*
Pitkäaho et al. (2015)	N	N	Y	Y	Y	Y	U	Y	Y	Include*
Potter et al. (2003)	N	N	N	N	U	Y	U	Y	Y	Include*
Roche et al. (2012)	N	Y	Y	Y	U	Y	U	Y	Y	Include*
Schneider et al. (2016)	N	Y	Y	Y	U	Y	U	Y	Y	Include*
Schreuders et al. (2015)	N	Y	Y	Y	Y	Y	U	Y	Y	Include*
Seago et al. (2006)	N	N	Y	Y	U	Y	U	Y	Y	Include*
Sochalski et al. (2008)	N	Y	Y	Y	U	Y	U	Y	Y	Include*
Staggs et al. (2016)	U	Y	Y	Y	Y	Y	U	Y	Y	Include*
Tzeng et al. (2011)	Y	Y	Y	Y	U	Y	Y	Y	Y	Include*
Yang (2003)	Y	U	Y	Y	Y	Y	U	Y	Y	Include*
Berkow et al. (2014)	N	Y	Y	Y	Y	U	U	Y	Y	Exclude§
Buchan & Dal Poz (2002)	-	-	-	-	-	-	-	-	-	Exclude§
Francis et al. (2005)	-	-	-	-	-	-	-	-	-	Exclude§
Griffiths et al. (2016)	N	N	Y	Y	U	Y	U	Y	Y	Exclude§
Hart, P. and N. Davis (2011)	N	Y	N	Y	Y	Y	U	Y	N	Exclude§
Hendrix, T. J. and S. E. Foreman (2001)	-	-	-	-	-	-	-	-	-	Exclude§
Huang et al. (2011)	N	Y	N	N	U	U	U	Y	U	Exclude§
Ibe et al. (2008)	N	N	N	Y	U	U	U	Y	N	Exclude§
Jacob et al. (2015)	-	-	-	-	-	-	-	-	-	Exclude§
Kenney, P. A. (2001)	n	y	u	y	n	u	u	y	n	Exclude§
Kutney-Lee, A. and L. H. Aiken (2008)	-	-	-	-	-	-	-	-	-	Exclude§
Lankshear et al. (2005)	-	-	-	-	-	-	-	-	-	Exclude§
Manojlovich et al. (2011)	N	N	N	Y	Y	U	U	Y	Y	Exclude§
McGillis Hall (2003)	Y	U	N	Y	N	Y	U	Y	U	Exclude§

Needleman et al. (2002) Policy highlights	-	-	-	-	-	-	-	-	-	Exclude[§]
Needleman et al. (2006)	-	-	-	-	-	-	-	-	-	Exclude[§]
Robinson et al. (2009)	-	-	-	-	-	-	-	-	-	Exclude[§]
Whiteman et al. (2002)	-	-	-	-	-	-	-	-	-	Exclude[§]

Y – Yes, N- No, U- Unclear

Descriptive/case series studies 2015 (9 questions): 1) Was study based on a random or pseudo- random sample? 2) Were the criteria for inclusion in the sample clearly defined? 3) Were confounding factors identified and strategies to deal with them stated? 4) Were outcomes assessed using objective criteria? 5) If comparisons are being made, was there sufficient descriptions of the groups? 6) Was follow up carried out over a sufficient time period? 7) Were the outcomes of people who withdrew described and included in the analysis? 8) Were outcomes measured in a reliable way? 9) Was appropriate statistical analysis used?

*Reason for inclusion:-Reported nurse sensitive outcome measures using statistical analysis

[§] Reasons for exclusion: Not skill mix; not patient outcome; narrative review; not research; doctor skill mix (not nurses); nursing home; nurse perception; small sample size; no data analysis; nursing outcomes (not patients)

Online supplement C4. Critical Appraisal results for included studies using the JBI - Experimental Studies 2015 Checklist

**Source**	**1**	**2**	**3**	**4**	**5**	**6**	**7**	**8**	**9**	**10**	**Decision**
Lee et al. (2005)	N	U	N	U	U	U	U	Y	Y	Y	Include*
Newhouse et al. (2013)	Y	N	U	U	U	Y	Y	Y	Y	Y	Include*
Twigg, D. et al. (2016)	N	U	U	Y	U	Y	Y	Y	Y	Y	Include*

Y – Yes, N- No, U- Unclear

Experimental Studies 2015 (10 questions): 1) Was the assignment to treatment groups truly random? 2) Were participants blinded to treatment allocation? 3) Was allocation to treatment groups concealed from the allocator? 4) Were the outcomes of people who withdrew described and included in the analysis? 5) Were those assessing the outcomes blind to the treatment allocation? 6) Were control and treatment groups comparable at entry? 7) Were groups treated identically other than for the named interventions? 8) Were outcomes measured in the same way for all groups? 9) Were outcomes measured in a reliable way? 10) Was appropriate statistical analysis used?

*Reason for inclusion:-Reported nurse sensitive outcome measures using statistical analysis
