# Supplementary material for: A quantitative systematic review of the association between nurse skill mix and nursing‐sensitive patient outcomes in the acute care setting
Source: J Adv Nurs. 2019 Oct 3;75(12):3404–23. doi: 10.1111/jan.14194 (PMC6899638; doi:10.1111/jan.14194)
Supplement: Supplementary file 4 [file JAN-75-3404-s004.pdf]

Online supplement D: Included Study Characteristics

First author (Yr)	Setting; Timeframe	Aim	Study Design; No. of participants	Definition of skill mix; Tool/ Instrument	Patient outcomes	General Findings
L. H. Aiken et al. (2016)	Acute care hospitals; 2009 - 2010	To determine the association of hospital nursing skill mix with patient mortality, patient ratings of their care and indicators of quality of care	Cross-sectional; Survey data Nurses:<br>- Hospitals: 243<br>- Nurses: 13,077<br>Survey data patients:<br>- Hospitals: 182<br>- Patients: 18,828<br>Discharge data:<br>- Hospitals: 188<br>- Patients: 275,519	Proportion (%) of professional nursing staff among total nursing personnel of all qualifications calculated for each hospital;<br>Data set analysis (Patient discharge data by hospital; hospital administrative information; AHRQ; HCAHPS survey	1) Patient mortality - measured as death in hospital within 30 days of admission, 2) Patient ratings of care, care quality, patient safety and adverse events.	Mean skill mix (% of professional nurses) was 65.6% ± 9.8%. Richer nurse skill mix (e.g. every 10-point increase in % of professional nurses among all nursing personnel) was associated with lower odds of mortality (OR=0.89), lower odds of reports of poor quality (OR=0.89), poor safety grades (OR=0.85) and other poor outcomes (0.80<OR<0.93), after adjusting for patient and hospital factors.
Ambrosi et al. (2016)	Acute internal medicine units; Patients over 65 years of age; 2011 - 2012 (7 months)	What is the in-hospital mortality rates among patients admitted to acute internal medicine units?	Secondary analysis of a longitudinal observational study<br>Units: 12<br>Total N: 1464 (N survival: 1364, N death: 100)	Proportion (%) of care offered from RNs. Ranged from 48% (less RNs; more NAs) to 78.9% (more RN care; minimal NA care);<br>Braden Scale for Predicting Pressure Ulcer Risk, Blaylock Risk Assessment Screening Score (BRASS), MISSCARE Survey	Mortality (% died in hospital)	Mean skill-mix for in hospital survival was 63.4% (69.2-63.9) and for in-hospital deaths 60.3% (58.1-62.0).<br>Patients 6x (RR 6.26 95 % CI = 3.632–10.794) more likely at risk of dying at weekends.<br>Patients receiving a higher skill-mix (more nursing care offered by RNs instead of NAs) at less risk of dying (RR = 0.940, 95 % CI = 0.912–0.969)
Anthony (2008)	Medical-surgical units; all adult patients who experienced hypoglycaemia; 3 month period	What is the relationship of RN staffing and adherence to practice guidelines, and patient outcomes for the treatment of hypoglycaemia?	Retrospective correctional design<br>Total:<br>N = 210<br>Hospital A: N=105<br>Hospital B: N=105	Total nursing department hours (including RNs, LPNs, NAs) per patient day HPPD, and the proportion filled by RNs (staff mix);<br>Review of patient notes in relation to hypoglycemia treatment and comorbidities	Total number of episodes of hypoglycaemia	Skill mix for Hospital A was positively related for hypoglycaemic patients, but not for Hospital B.

First author (Yr)	Setting; Timeframe	Aim	Study Design; No. of participants	Definition of skill mix; Tool/ Instrument	Patient outcomes	General Findings
Aydin et al. (2015)	Medical-surgical units; Jan 2009 - Apr 2010	What is the impact of nursing on falls, injury falls (moderate or greater), from falls and restraint, as a risk mitigation intervention?	Multivariate study testing predictive models Units: 789	% RN HPPD (73.76 ± 10.41; IQR: 11.43); LVN % Licensed HPPD (3.05 ± 5.46; IQR: 4.38); % un-licensed HPPD (23.20 ± 9.30; IQR: 10.86); Sitter hours as a percentage of total care hours (4.05 ± 6.02; IQR: 4.98); Data set analysis (CalNOC)	1) Falls and injury falls (moderate or greater), 2) restraint	Mean skill mix: 73.8% ± 10.41 (IQR: 11.43). Increasing skill mix resulted in an improvement in patient safety and injuries. Fewer falls/injury falls were predicted by more unlicensed care hours. Lower restraint use was predicted by more RN hours and more certified RNs. Falls/restraints outcome variables (n = 721 units): Falls per 1000 patient days (mean): 3.21 C 1.62; IQR: 1.91. Injury falls per 1000 patient days (moderate and greater) (mean): 0.09 ± 0.15; IQR: 0.15. Units with a 1-SD increase (9.3%) in the % of care hours delivered by unlicensed staff were twice as likely (odds ratio of excess zeroes, 2.0) to have no injury falls. One-SD increase (10.41%) in percentage of care hours delivered by RNs is estimated to decrease percentage of patients in restraints by 15%.
Bae et al. (2014)	Acute care units; Oct 2010 - Mar 2012	Explore the association between comprehensive nurse staffing characteristics and patient falls and pressure ulcers	Retrospective observational design Hospitals: 3 Units: 35	Proportion of RNs to LPNs and UAPs; Data analysis (Review of nursing-sensitive quality indicators and nurse staffing data)	1) patient falls, 2) patient falls with injuries 3) pressure ulcers (both total pressure ulcers and unit-acquired pressure ulcers)	Mean monthly staffing levels were 8.23 HPPD for RNs. Rates of patient falls and injury falls higher with higher temporary RN staffing levels, but decreased with greater levels of LPN care HPPD. Pressure ulcers were not related to any staffing characteristics. 1 hour increase in LPN care HPPD led to decreased in-patient falls by a factor of 0.540 (monthly, $P < .05$). Units that used temporary RN staff to provide equal to or more than 0.3 care HPPD had a rate 1.552 times greater for patient falls (monthly, $P < .01$). Units with greater than zero but less than 0.3 care HPPD provided by temporary RN staff had a rate 4.169 times greater for injury-related falls (quarterly, $P < .05$).
Ball et al. (2018)	Acute care hospitals; 2007 - 2010	Examine if missed nursing care mediates the observed association between nursing staffing levels and mortality.	Retrospective cohort study Hospitals: 9 N: 422,730	Nurse staffing (RN: Patient ratio), Nurse Education: % with degree; Data analysis (administrative data on discharge status); Nurse Survey	30-day in-patient mortality (mediated by missed care)	Mean RN patient ratio: 8.3 ± 2.4. Each additional patient per nurse associated with 7% increase in odds of a patient dying within 30 days of admission (OR 1.068 95% CI 1.031–1.106, p=0.0002). Every 10% increase in bachelor's degree nurses associated with decrease in 30-day mortality by 7% (OR 0.929, 0.886–0.973, p=0.0019).

First author (Yr)	Setting; Timeframe	Aim	Study Design; No. of participants	Definition of skill mix; Tool/ Instrument	Patient outcomes	General Findings
Barkell et al. (2002)	Surgical unit; Postoperative bowel procedure patients; Jan 1999 - Jun 2000	Examine the effects of a change in the staffing model on length of stay, variable cost, patient satisfaction, incidence of UTI and pneumonia, and pan management in bowel resection patients.	Retrospective, descriptive comparison design Staffing model A: 59 patients Staffing model B: 37 patients	% of RNs of total caregiver staff; Medical record review; Parkside Patient Satisfaction Survey	1) LOS, 2) incidence of pneumonia 3) incidence of UTI 4)pain	Staffing model A (RNs 25.5 FTE & PCAs 13.3 FTE) vs Staffing model B (RNs 23.8 FTE & PCAs 6.5 FTE) (PCAs: Patient care associates). Significant differences in pain management outcomes between staffing models. Patients' perception of pain as measured by mean pain scores for post-op days 1 and 2 statistically significant (P = .017) (1.9 vs 2.6). Mean number of pain scores documented by nurses decreased slightly in staffing model B and was statistically significant (7.5 vs 6.0; P = .006).
Blegen et al. (2011)	General and ICUs; 2005	Determine: relationship between nurse staffing in general and intensive care units and patient outcomes; whether safety net status affects this relationship.	Cross sectional administrative datasets 1.1 million adults across 872 adult inpatient units (285 ICUs, 587 general)	Proportion of hours per day (TotHPD) provided by RNs. Total hours of care from RNs, LPNs, and UNAs for each patient day (TotHPD). RN Skill mix: proportion of those hours that were provided by RNs; Review of clinical and operational data sets	1) in-hospital CHF mortality, 2) Decubitus ulcer, 3) FTR, 4) infections due to medical care, 5)postoperative sepsis, 6) rate of LOS	The proportion of the total hours delivered by RNs averaged 60% in general units and 76% in ICUs. RN skill mix in general units was associated with reduced FTR (p<0.01) and infections (p<0.05), and in ICU with fewer cases of sepsis (<0.01) and FTR (p<0.05)
Bolton et al. (2007)	Medical-surgical and step-down units; more than 500,000 patient days over 3 years; 2002 - 2006	What is the impact of mandatory nursing ratios of nursing quality in acute care hospitals?	Follow-up and extension to previous analysis Total units: 252: (187 medical-surgical units; 65 step-down units)	Pre Regulated Data 2002 minimum staffing requirements: Patients: 1:2 & Step-down patients: 1:4 Mandated nurse ratios were 1:6 (nurse to patients) in 2004 and 1:5 from 2005 onwards; Data set analysis (CalNOC); Pressure ulcer and restraint data review	1) Falls, 2) pressure ulcers	Skill mix data reflected overall reductions in the use of LVNs in both medical-surgical (decreased from 8% to 6%) and stepdown units (from 4% to 2%) over the 4-year observation period. % of care provided by RN staff increased significantly from 2002 to 2006 in medical-surgical units by 11.2%. Significant negative association between % of contracted staff & falls with injury (coefficient = −0.003, p = .008, medical-surgical units). Significant negative association between % of care hours provided by RN staff and falls (coefficient = −0.02897, p = .008, stepdown).

First author (Yr)	Setting; Timeframe	Aim	Study Design; No. of participants	Definition of skill mix; Tool/ Instrument	Patient outcomes	General Findings
Boyle et al. (2016)	Patient care units; 2013	Develop a unit-level inpatient composite nursing care quality performance index - the Pressure Ulcer and Fall Rate Quality Composite Index.	Two-phase measure development study<br>Hospitals: 857<br>Units: 5144	Total nursing HPPD and nursing skill mix;<br>Pressure Ulcer and Fall Rate Quality Composite Index	Composite quality indicator combining pressure ulcer rate and total fall rate (Pressure Ulcer and Fall Rate Quality Composite Index)	Nursing skill mix percentage ranged from 59% in rehabilitation units to 84% in critical care units. Lower total nursing HPPD (B = 0.101, p = 0.001), higher registered nurse skill mix (B = 0.018, p = 0.018), higher % of registered nurses with baccalaureate or higher degree (B = 0.009, p = 0.010), higher % of registered nurses with national specialty certification (B = 0.010, p = 0.014), lower % of nursing hours supplied by agency nurses(B =0.066, p = 0.003) were each significantly associated with higher Pressure Ulcer and Fall Rate Quality Composite.
Breckenridge-Sproat et al. (2012)	Army hospital: Medical-surgical, step-down, and critical care;<br>2003 - 2006	What are the associations of nurse staffing and workload on Army hospital units?	Secondary analysis of a longitudinal data set<br>Hospitals: 4<br>Units: 23	% of the total nursing care hours that were worked each shift by RNs, LPNs, or NAs; Data set analysis (MilNOD)	1) Medication errors, 2) falls	The skill mix mean for RN on medical wards was 0.51 (±0.06), on step-down was 0.59 (±0.08) and on critical care was 0.80 (±0.12). A higher % of LPNs is associated with a higher medication error rate 3.807, $p < 0.05$. RN skill mix is not a statistically significant predictor of medication errors or falls.
Chang et al. (2011)	Medical-surgical or medical-surgical specialty units;<br>2003 - 2004 (6 months)	Is the relationship between error-producing conditions and medication errors, moderated by the learning climate?	Cross-sectional descriptive study<br>Hospitals: 146<br>Units: 279<br>N = 2,744	% of nursing care hours delivered by RNs; care hours delivered by all nursing personnel (RNs; LPNs; LVNs; nurse aides); Staff questionnaires, incident reports	1) Medication errors	62% of nursing care hours were provided by RNs. There were no significant outcomes in the relationship between skill mix and medication errors. When learning climate was negative, having more RNs was associated with fewer medication errors.
Cho et al. (2003)	Acute care hospitals;<br>1996 - 1999	What are the effects of nurse staffing on adverse events, morbidity, mortality, and medical costs?	Cross-sectional descriptive study<br>Units: 232<br>N = 124,204	RN proportion and RN Hours (total productive hours by registered nurses per patient day) divided by All Hours (total productive hours worked by all nursing personnel per patient day) compared to all Hours (total productive hours worked by all nursing personnel per patient day); Data set analysis (OSHPD Hospital Financial Database; SID)	1) Patient fall/injury, 2) pressure ulcer, 3) adverse drug event, 4) pneumonia, 5) UTI, 6) wound infection, 7) sepsis	There is a significant inverse relationship between RN hours and RN proportion, and pneumonia. An increase of 1 RN Hours resulted in an 8.9% (OR: 0.911) decreased chance of pneumonia. On average, the probability of pneumonia was 0.23% lower for a 1-hour increase in RN Hours, decreasing the overall pneumonia rate from 2.59% to 2.36%.

First author (Yr)	Setting; Timeframe	Aim	Study Design; No. of participants	Definition of skill mix; Tool/ Instrument	Patient outcomes	General Findings
Choi et al. (2014)	Acute care units; 2011	What is the relationship between 6 nurse staffing measures and UAPUs?	Descriptive, correlational study<br>Hospitals: 409<br>Units: 2397<br>Total RNs = 57,223	Proportion of total nursing hours provided by RNs compared to total nursing HPPD and non-RN HPPD; Data set analysis (NDNQI®); RN survey	UAPUs	The mean RN HPPD was 8.57 (±4.22). An increase of 1 percentage point in the RN mix was associated with an estimated 1.2% reduction in the UAPU odds.
de Cordova et al. (2014)	Medical, medical/ surgical, surgical, step-down, telemetry units in VA hospitals;<br>Oct 2002 - Dec 2006	What is the relationship between RN staffing levels, skill mix, and experience on the night shift, in comparison to LOS?	Longitudinal descriptive study<br>Hospitals: 138<br>Units: 185<br>N = 8243	Skill mix day shift (RN, LPN, UAP), compared to each group. For each personnel type, an average HPPD variable was created for each month divided by patient bed days; Data set analysis (administrative and electronic data)	LOS	The percent of HPPD provided by UAPs during the day and the presence of larger proportion of UAPs in relation to RNs, were associated with longer LOS ($\beta = 0.20$, $p < 0.01$). The percent of hours worked by UAPs at night increased (and the percent of hours worked by licensed nurses went down), and the LOS increased ($\beta = 0.18$, $p = 0.02$). For each hour, staffing mix was lower on nights relative to the day shift, and the LOS increased by 1.5%.
Donaldson et al. (2005)	Acute care Hospitals; Observation step-down and medical/ surgical units;<br>2002-2004	What is the impact of mandated minimum-staffing ratios on nursing hours of care and skill mix?	Prevalence study (cross-sectional study)<br>Hospitals: 68<br>Units: 268<br>Step-down units: 68 Medical and surgical units: 200<br>Total patient days: Approx 196,000	% of RN hours from total nursing-care hours, compared to the percent of LVN nursing-care hours from total nursing care hours, and the % of all non-RN and LVN nursing-care hours from total nursing care hours<br>Data set analysis (CalNOC); Pressure ulcer prevalence studies	1) Patient falls,<br>2) pressure ulcers	Medical and surgical had a skill mix of RN hours of 59.2% pre and 66.7% post. Step down had a skill mix of RN hours 68.8% pre and 72.2% post. Mean total RN hours of care per patient day increased by 20.8% on medical and surgical units. No significant changes were found despite research linking nurse staffing with fall rates and pressure ulcers.
Duffield et al. (2011)	Acute care units; 2001-2006 (fiscal years)	What is the relationship between nursing skill mix, nursing workload, nursing work environment, and patient outcomes?	Longitudinal retrospective, and cross-sectional<br>Longitudinal data:<br>Hospitals: 27<br>Units: 286<br>Nurse roster/payroll records: 10,963,806<br>Cross-sectional data:<br>Hospitals: 19<br>Units: 80	% of RNs on unit, compared to the percent of CNSs, ENs, AINs, and TENs;<br>Data set analysis (HIE and AHS databases); NWI-R Nurse survey	11 OPSN (UTI, pressure ulcers, pneumonia, DVT/pulmonary embolism, ulcer/GI bleeding, central nervous system complications, sepsis, shock/ cardiac arrest, surgical wound infection, pulmonary failure, physiological/ metabolic derangement)	Mean total nursing time 68.4% RN, 7.4% CNS, 20.4% EN and 3.8% AIN/TEN. An increase in the RN/CNS hours was associated with significant ($p \leq 0.01$) decreases in six OPSN: decubiti, GI bleeding, physiological/metabolic derangement, pulmonary failure, sepsis, and shock.

First author (Yr)	Setting; Timeframe	Aim	Study Design; No. of participants	Definition of skill mix; Tool/ Instrument	Patient outcomes	General Findings
Esparza et al. (2012)	Medical-surgical units in acute care hospitals; 2006	What is the relationship between RN amount and staffing and skill mix patterns?	Cross-sectional study<br>Hospitals: 253<br>Over 2 million patient discharges analysed	% of RN hours relative to total care hours & the mandated nurse-patient ratio, compared to the proportional skill mix of productive hours provided by each type of nursing caregiver relative to the total nursing HPPD (LVNs; NAs); Data set analysis (OSHPD, HADR); Patient Level Discharge Database (PLDD)	1) UTI, 2) LOS	The mean medical-surgical Nursing HPPD was 9.417 (range 3.54-16.98), and the mean proportion of RNs was 0.692 (0.311-1.00). As the RN proportion of skill mix increased, the OR of UTI decreased by 4.25 times ($p < 0.001$) (CI 3.409 – 5.299). The higher the proportions of the RN skill mix, the shorter the LOS ($p < 0.001$; only 0.1% of the variation in LOS).
Estabrooks et al. (2005)	Acute care hospitals; Admitted for acute MI, CHF, chronic obstructive pulmonary disease, pneumonia or stroke; Sep 1998 - Feb 1999	What is the effect of nursing characteristics in predicting a 30-day mortality rate in hospitals?	Cross-sectional analysis<br>Units: 49<br>Patients discharged: 18,142	RN compared to total nursing staff, compared to non-RN compared to total nursing staff; Analysis of hospital discharge abstracts and vital statistics; NWI-R Nurse survey and Maslach Burnout Inventory	1) 30-day patient mortality (following acute MI, CHF, COPD, pneumonia, stroke)	The mean skill mix was 0.66 ±0.42.<br>Hospitals with a higher proportion of registered nurses (i.e. higher RN-to-non-RN ratios) were associated with lower rates of 30-day patient mortality. OR: 0.83 (0.73 – 0.96) for richer nurse skill mix; 1.26 (1.09 – 1.47) for higher proportion of casual or temporary positions. Significant nursing characteristics that predict 30-day mortality were as follows: 0.81 (0.68–0.96) for higher nurse education level, 0.83 (0.73–0.96) for richer nurse skill mix, 1.26 (1.09–1.47) for higher proportion of casual or temporary positions.
Frith et al. (2010)	Medical-surgical units; Jul 2005 - Jun 2007	What are the effects of nurse staffing on hospital-conditions and LOS?	Multisite, cross-sectional retrospective study<br>Hospitals: 4<br>Units: 11<br>Patient cases: 34,838	% of RN staff on the units, compared to the % of LPNs; Data set analysis (administrative databases)	1) LOS, 2) adverse events (catheter-associated UTIs, pressure ulcers, hospital-acquired injuries, air embolism, blood incompatibilities, vascular catheter–associated infections, and mediastinitis after coronary bypass graft)	The mean RN skill mix was 45.5% ± 5.31 (range: 35%-59%). The % of RNs to adverse events decreased −0.034467 ($p < 0.01$); the % of LPN staff was not significant. Both RN and LPN percentages are significantly negatively related to shorter patient LOS (percent RN staff: −0.030088 ($p < 0.01$); percent LPN staff: −0.027216 ($p < 0.01$)). A higher percentage of RNs in the skill mix predicted a lower number of adverse events and shorter LOS. An increase of 1% in RN percentages in staffing reduced the number of adverse events by 3.4%, and a 5% increase in the RN % would decrease the number of adverse events by 15.8%.

First author (Yr)	Setting; Timeframe	Aim	Study Design; No. of participants	Definition of skill mix; Tool/ Instrument	Patient outcomes	General Findings
Glance et al. (2012)	Trauma centres; 2006	What is the association between nurse staffing and hospital outcomes in injured patients?	Cross-sectional study<br>Admitted to 77 Level 1 and Level 2 centres<br>Patients: 70,142	The change in LPN staffing levels; the bottom quartile of LPN HPPD, compared to the top quartile of LPN HPPD;<br>Data set analysis (2006 HCUP, NIS and AHA Annual Survey)	1) Mortality, 2) Healthcare associated infections (HAI), 3) FTR	Proportion of total nurse staffing hours' time for RNs the median was 96.9% (IQR: 95.0-98.4) and for LPNs the median was 3.1% (IQR:1.6-5.0). A 1% increase in the ratio of LPN to the total nursing time is associated with a 4% increase in the odds of mortality (adj OR 1.04; 95% CI: 1.02-1.06; p = 0.001) and a 6% increase in the odds of sepsis (adj OR 1.06: 1.03-1.10; p < 0.001). Hospitals in the highest quartile of LPN staffing had 3 excess deaths (95% CI:1.2, 5.1) and 5 more episodes of sepsis (95% CI: 2.3, 7.6) per 1000 patients compared to hospitals in the lower quartile of LPN staffing.
Goode et al. (2011)	Magnet & Non-Magnet acute care hospitals; 2005	What is the relationship between staffing and patient outcomes in Magnet and non-Magnet hospitals?	Bivariate and multivariate analyses<br>Hospitals:<br>19 Magnet hospitals<br>35 Non-Magnet Hospitals	RN staffing mix %for Magnet hospitals, compared to RN staffing mix % for Non-Magnet hospitals; Data set analysis (UHC operational and clinical databases)	1) mortality rates CHF and MI, 2) FTR, 3) HAPU, 4) infections, 5) postoperative sepsis, 6) LOS	Non-Magnet hospitals had a 2% higher RN skill mix than Magnet hospitals (General units 61% compared to 58% and ICU 77% compared to 75%. Staffing on adult ICUs: RN% FTR -0.010 (p < 0.05), Sepsis -0.033 (p < 0.05), CHF Mortality 0.014 on ICU only (p < 0.05). Staffing on adult general units: RN% FTR -0.010 (p < 0.05).
He et al. (2016)	Participating acute care NDNQI hospitals; 2004-2012	What is the longitudinal relationship between nurse staffing and patient outcomes, such as falls and hospital-acquired pressure ulcers?	Longitudinal study<br>Inpatient falls:<br>Hospitals: 1622<br>Units: 1240<br>Total falls: 13,339<br>Pressure ulcers:<br>Hospitals: 1527<br>Units: 848<br>Total ulcers: 12,435	RN skill mix (% of nursing hours provided by RNs), compared to the total nursing HPPD by all staff; Data set analysis (NDNQI®)	1) falls, 2) HAPU	The mean RN skill for falls was 0.7 and for pressure ulcers was 0.8. RN skill-mix was positively associated (higher staffing related to higher event rate) with fall rate (p < 0.001) and HAPU rate (p = 0.03), inversely associated with the rate of pressure ulcers stage III or above (p < 0.001), and not associated with injurious fall rate (p = 0.08).
He et al. (2013)	Acute care units in VA medical centers; inpatient admissions with LOS ≤ 90 days; Oct 2007 - Sep 2008	What is the impact of patient-level risk adjustment on the associations of unit level<br>nurse staffing and 30-day inpatient mortality?	Retrospective cross-sectional study<br>Hospital: 128<br>Units: 446<br>Hospitalisations: 284,097	RN skill mix = Total hours supplied by RNs, compared to total nursing productive HPPD (total HPPD); Review of hospital discharge records	1) 30-day inpatient mortality	The mean RN skill mix for ICU was 0.91 ± 0.09 and for non-ICU was 0.57 ± 0.11. For non-ICU, higher RN skill mix was associated with lower mortality risk (p < 0.05). RN skill mix (10 percentage points): Low: OR 0.93 (95% CI 0.88 - 0.99) p = 0.02; Moderate: OR 0.94 (95% CI 0.90 - 0.99) p = 0.01; High: OR 0.96 (95% CI 0.93 - 1.00) p = 0.05

First author (Yr)	Setting; Timeframe	Aim	Study Design; No. of participants	Definition of skill mix; Tool/ Instrument	Patient outcomes	General Findings
Huston (2001)	Surgical units; the population had a discharge DRG 209 of major joint and limb reattachment procedures of the lower extremity; Jan - Mar 1996 & 1997	What are the correlations between changing staffing mix and postoperative pain management?	Retrospective descriptive study Hospitals: 2 Units: 2 Patients: 203	% of RNs with direct patient care responsibilities. Staffing model change from a traditional team(leading model RNs, LVNs, and UAP prior to 1996) compared to new staffing model (increased UAPs and decreased RNs); Retrospective random chart review; patient survey (standardized numeric pain scale data collection instrument)	Pain	Correlation between mean NAA pain scale scores and UAP staffing r = 0.52, suggested a fair degree of relationship. Correlation between mean NAA pain scale scores and RN staffing r = -0.32, suggested a slight degree of negative correlation. No difference in pain during the 1st quarter of 1996 and the 1st quarter of 1997 and mean RN staffing.
Johansen et al. (2015)	ED; Patients aged 18-90 years with symptoms of ACS; Jan 1 2008 - Jan 31 2010	What is the effect of nurse resources on the process of care in all New Jersey hospital-based emergency departments?	Secondary analysis of ED data Hospitals: 73 Patients: 1343	Proportion of all licensed nurses (RNs, LPNs, and aides) that were RNs; Data set analysis (ED patient data, ED nurse staffing data; hospital characteristic data)	Care processes for ACS or acute MI: aspirin on arrival and percutaneous coronary intervention within 90 minutes of hospital arrival	RN skill mix mean was 0.71 ± 0.08. The skill mix in some EDs, and an increase in the number of patients assigned to a nurse, might prevent patients with ACS or acute MI from receiving lifesaving care. Each 10% increase in the proportion of RNs is significantly associated with a 7.1% increase in aspirin on arrival and a 6.3% decrease in percutaneous coronary intervention within 90 minutes of hospital arrival.
Kim et al. (2016) (a)	Acute care hospitals; COPD; 2002 - 2012	Evaluate the effects of nursing staffing on hospital readmission of COPD patients	Retrospective observational study Hospitals: 1,070 n=339,379 n=338,369	Number of RNs per 100 beds and the proportion of RNs on staff to one of three groups (Q1: low; Q2: moderate; Q3: high); Data set analysis (NHI claim data)	Readmission to hospital within 30 days after discharge for COPD.	Higher proportion of RNs significantly associated with lower readmission rate, with 9.6% (Q2) and 22.6% (Q3) of reduction observed, compared with Q1 (Q2: OR= 0.90, p=.013; Q3: OR= 0.77, p < .001). An increased number of RNs per 100 beds was significantly associated with a lower readmission rate, with 13.0% (Q2) and 11.3% (Q3) of reduction observed, compared with Q1 (Q2: OR = 0.87, p < .001; Q3: OR= 0.89, p=005)
Kim et al. (2016) (b)	Acute care hospitals; Patients aged 20-85 years who underwent hip or knee surgery and not admitted to ICU; 2009 - 2010	What is the relationship between nurse staffing levels on the length of stay and medical expenses of patients who underwent hip or knee surgeries?	Cross-sectional study Hospitals: 222 Patients: 22,289	RN proportion to nursing staff which were < 91.4, compared to the RN proportion to nursing staff which were > 91.4; Data set analysis (NHI database and NHI Corporation hospital survey data)	LOS of hip/knee surgery patients	Maintaining a high nurse staffing level could be a cost-effective strategy. When number of beds per RN increased by 1, LOS significantly increased by 0.7 days (model 2). Proportion of registered nurse ratio <91.4, N 11,109, mean LOS 21.1 (SD 10.1), 91.4+, N 11,180 Mean LOS 18.3 (SD 10.8) p<0.001. Hospitals with a median or higher bed-to-nurse ratio had an LOS of 4.89

First author (Yr)	Setting; Timeframe	Aim	Study Design; No. of participants	Definition of skill mix; Tool/ Instrument	Patient outcomes	General Findings
						days longer than those with lower than the median bed-to-nurse ratios.
Kim et al. (2018)	Tertiary hospitals; 2013 - 2014	Describe the status and prove the relationships of nurse staffing level with nursing sensitive outcome indicators for adult medical and surgical inpatients	Retrospective observational study<br>Hospitals: 46<br>n=338,369	Grades are determined by the ratio of beds to RNs (bed-to-nurse ratio) - calculated by dividing total number of beds by total number of fulltime equivalent RNs working in the general ward. Assistive personnel and vocational/ practical nursing license nurses were excluded;<br>Nurse staffing grades 1) bed-to-nurse ratio < 2:1, 2) ranging from 2:1 to less than 2.5:1 and 3) 2.5:1 to less than 3:1;<br>Data set analysis (HIRA)	1) UTI, 2) upper GI tract bleeding, 3) DVT, 4)hospital-acquired pneumonia,<br>5) pressure ulcer,<br>6) sepsis,<br>7) shock/cardiac arrest, 8) CNS complication, 9) in hospital death,<br>10) wound infection,<br>11) physiologic/ metabolic derangement and 12) pulmonary failure	6 NSOs (UTI (grade 2 - AOR 2.17 [CI 2.08-2.26], grade 3 2.41 CI[2.30–2.52]), upper GI tract bleeding (grade 2 -1.60 CI (1.54–1.67), grade 3 1.80 (1.72–1.89), hospital-acquired pneumonia (grade 2 1.25 (1.21–1.28), grade 3 1.32 (1.27–1.36), shock/cardiac arrest (grade 2 1.77 (1.69–1.85), grade 3 2.44* (2.32–2.57), in-hospital death (grade 2 1.08 (1.04–1.11), grade 3 1.17* (1.12–1.21) , and wound infection (grade 2- 1.88 (1.70–2.08), grade 3 2.23* (1.99–2.50) (all $p < 0.0001$)) increased as nurse staffing level decreased even after adjusting for patient and hospital characteristics. 5 NSOs - DVT, physiologic/metabolic derangement, CNS complication, pulmonary failure and sepsis - showed weak relationships with nurse staffing grade after adjusting for patient and hospital characteristics. Statistically significant associations between higher nurse staffing level and rates of 11 NSOs (not pressure ulcer).
Lake et al. (2010)	Magnet and non-Magnet hospitals; 2004	What is the relationship between nursing unit staffing, Magnet status, and patient falls?	Retrospective cross-sectional observational study<br>Hospitals: 636<br>Magnet: 108<br>Non-magnet: 528<br>Units: 5,388<br>Patient falls: 113,067	RN HPPD, compared to non-RN HPPD LPN & NA;<br>Data set analysis (NDNQI®; AHA 2004 Annual Hospital Survey; Medicare CMI, hospital's Magnet status)	Patient fall (with or without injury)	The mean RN HPPD for ICU was 14.84, stepdown was 7.03, medical was 5.11, surgical was 5.22, medical-surgical was 5.04 and rehabilitation was 4.02. Patient safety may be improved by creating environments consistent with Magnet hospital standards. RN HPPD is negatively associated with fall rate; conversely, LPN and NA HPPD were positively associated with fall rate: $r = -0.29$ for RN HPPD; $r = 0.12$ for LPN HPPD, and $r = 0.10$ for NA HPPD ($p < 0.001$). An additional RN HPPD was associated with a 3% lower fall rate in ICUs. An additional LPN or NA hour was associated with a 2–4% higher fall rate in non-ICUs.

First author (Yr)	Setting; Timeframe	Aim	Study Design; No. of participants	Definition of skill mix; Tool/ Instrument	Patient outcomes	General Findings
Leary et al. (2016)	Acute care hospital; 9 years	Determine the relationship between registered and non-registered nurse staffing levels and clinical outcomes could be discovered through mining of routinely collected data.	Descriptive correlational design Hospital: 2 Units: 33	Staffing levels for RNs and unregistered HCSW; Data set analysis (clinical data extracted and mined against staffing and outcomes database using Mathmatica V.10.)	1) Falls, 2) pressure ulcers	Wards with a higher ratio of RN to HCSW appear to have less falls. Total nurses (whole time equivalent) highly correlated with falls rho 0.49 p value 3.6×10^{-12}. No significant correlation between staffing and pressure ulcers.
Lee et al. (2005)	General hospital; 2003	Examining the outcome of personnel cost and quality of care after implementing the skill mix practice model for nursing.	Pre- and post-test quasi-experimental design Hospitals:1 n=100	Skill mix practice model for nursing defined as practice of using both nurses and NAs to carry out nursing activities; Structured questionnaires (patients and staff)	1) Falls, 2) medication error rate	No specific findings to skill mix. Fall rate and medication error rate showed no statistically significant variation, but nursing education rate indicated a rising trend.
Martsolf et al. (2014)	Acute care, state hospitals; 2008 - 2011	Effect of nurse staffing on quality of care and inpatient care costs.	Retrospective longitudinal study Hospitals: 421 n=18,474,860	Total nursing staff (licensed + aides) per 1000 patient days (staffing changes 2008-2011); Data set analysis (state hospital financial and utilization reports)	1) LOS, 2) adverse events defined by 8 NS indicators (death in low mortality diagnosis related groups, death rate amongst surgical inpatients with serious treatable complications, central line catheter related blood stream infection, post-op respiratory failure rate, peri-op pulmonary embolism or DVT rate, post-op sepsis rate, post-op urinary complications, post-op pneumonia)	The mean nursing staff that are licensed nurses was 81.49 ± 7.97. A higher staff skill mix did not result in changes in adverse events or LOS.

First author (Yr)	Setting; Timeframe	Aim	Study Design; No. of participants	Definition of skill mix; Tool/ Instrument	Patient outcomes	General Findings
McCloskey et al. (2005)	Acute care, public hospitals; Jul 1993 - Dec 2000	Examine the effects hospital reengineering may have on adverse patient outcomes and the nursing workforce.	Retrospective longitudinal study Hospitals: 85 N= 3.3 million inpatient discharges; N=65,221 nurse responses	% of total nursing FTEs who were RNs; Data set analysis (NMDS, NWD)	11 NSOs (CNS complications; decubitus ulcers; DVTs and pulmonary emboli (PE); pneumonia; sepsis; shock and cardiac arrest; upper GI bleeding; UTI; pulmonary failure; physiologic and metabolic derangement; and surgical wound infections) average LOS and mortality.	After 1993, nursing FTEs and hours decreased by 36% and skill mix increased 18%. Substantial increase in many of the adverse clinical outcomes rates after reengineering's implementation, a simultaneous decrease in ALOS, and decreasing or stable mortality rates. Changes in the nursing workforce variables explained approximately 50% to 80% of the variance in CNS complications, decubitus ulcers, and sepsis rates among medical discharges and 50% to 96% of the variance in CNS complications, decubitus ulcers, DVT/PE, sepsis, UTI, physiological and metabolic derangement.
McGillis Hall et al. (2004) (a)	Medical, surgical and obstetric patient care units; **Timeframe not supplied**	Evaluate the effect of different nurse staffing models on costs and the patient outcomes of patient falls, medication errors, wound infections, and UTI.	Descriptive correlational design Hospitals: 19 Units: 77	1) RN/RPN staff mix, 2) all-RN staff mix, 3) proportion of regulated to unregulated staff, and 4) RN/RPN/ unregulated staff mix; Questionnaires and review of administrative records	1) Patient falls, 2) medication errors, 3) wound infections, and 4) UTIs	The lower the proportion of professional nursing staff employed on a unit (RNs/RPNs), the higher the number of medication errors and wound infections. Proportion of Professional staff - Medication Errors, T -3.25 P <0.05, Adj R2 = .37, df = (5, 17); Wound Infections, T -2.57 P< 0.01, Adj R2 = .49; df = (5, 17)
McGillis Hall et al. (2004) (b)	Medical, surgical and obstetric patient care units; **Timeframe not supplied**	Explore whether nurse staffing models and nursing demographic variables explain variations in quality outcomes.	Descriptive correlational design Hospitals: 19 Units: 77	1) Proportion of regulated to un-regulated workers (URW), 2) RN/RPN mix, 3) All RN mix, 4) RN/RPN/URW mix; Questionnaires and review of administrative records	Quality outcome data (on perceptions of quality of care, overall unit communication, and coordination of care)	All RN staff model had statistically significant positive relationship on nurses' perceptions of quality of care provided on unit (t = 2.43 p<.05).

First author (Yr)	Setting; Timeframe	Aim	Study Design; No. of participants	Definition of skill mix; Tool/ Instrument	Patient outcomes	General Findings
McGillis Hall et al. (2003)	Teaching hospitals; **Timeframe not supplied**	Evaluate the impact of different nurse staffing models on the patient outcomes of functional status, pain control and patient satisfaction with nursing care.	Repeated-measures study Hospitals: 19 n=2046	Nurse staff-mix variable was a categorical variable with 3 levels (all RN, RN/RPN, and RN/unregulated); Questionnaires (FIM - data collector/staff member, SF-36 - patients)	Change in health outcomes over time - pain control, outcomes of functional status: 1) simple pneumonia, 2) COPD, 3) major intestinal and rectal surgical procedures, 4) major noncancerous-related gynaecologic and uterine procedures, and 5) vaginal deliveries	Proportion of regulated nurses associated with better FIM instrument scores at hospital discharge (FIM t = 2.90, p<.01). Staff mix included RNs and unregulated workers associated with better pain outcomes at discharge than a mix that involved RNs/RPNs and unregulated workers (SF36 t= 2.28 p<.05). No evidence that nurse staffing variables and staff-mix variables influenced medical-surgical patient outcome at 6 weeks post-discharge. None of the nurse staffing variables were significant predictors of the pain reported obstetric patients after discharge. No evidence that nursing variables influenced functional adjustment after delivery.
Needleman et al. (2002)	Acute care, non-federal hospitals; Jul 1997 - Jun 1998	Examined the relation between the level of staffing by nurses in hospitals and rate of adverse outcomes among patients.	Descriptive correlational design Hospitals: 799 N=5,075,969 discharges of medical patients N= 1,104,659 discharges of surgical patients	Mix of skills and proportion of hours of care by licensed nurses (RN–hours plus LPN–hours); Data set analysis (OSHPD, AHA Annual Survey of Hospitals)	14 adverse outcomes: 11 for both medical and surgical patients (LOS, UTI, Pressure ulcers, Hospital-acquired pneumonia, Shock or cardiac arrest, Upper GI bleeding, Hospital-acquired sepsis, DVT, CNS complications, In-hospital death, FTR); 3 for surgical patients only (Wound infection, Pulmonary failure, Metabolic derangement)	A higher proportion of hours of nursing care provided by RNs and a greater number of hours of care by RNs per day were associated with better care for hospitalized patients. Proportion of RN-hours (medical patients): LOS -1.12 (-2.00 to -0.24) p=0.01, UTIs 0.48 (0.38 to 0.61) p<0.001, Upper GI bleeding 0.66 (0.45 to 0.96) p=0.03, pneumonia 0.59 (0.44 to 0.80) p=0.001, shock or cardiac arrest 0.46 (0.27 to 0.81) p=0.007, and FTR 0.81 (0.66 to 1.00) p=0.05. Proportion of RN-hours (surgical patients): 0.67 (0.46 to 0.98) p=0.04

First author (Yr)	Setting; Timeframe	Aim	Study Design; No. of participants	Definition of skill mix; Tool/ Instrument	Patient outcomes	General Findings
Newhouse et al. (2013)	Acute care hospitals; heart failure; Jul 2007 - Mar 2009	To evaluate the effect of a rural hospital quality collaborative and organisational context.	RCT - crossover. Phased cluster-randomized trial with pre-intervention, baseline and post-intervention measures. Hospitals: 23	Number of hours worked by each type of nursing staff [RN, LVN/LPN, UAP, and contract] (HPPD); Data set analysis (hospital data systems); written Teleform survey; PES	Heart failure core measures: lower compliance with discharge instructions, smoking cessation counselling, compliance with left ventricular ejection fraction assessment, prescribing angiotensin converting enzyme inhibitors on discharge	Nursing skill mix group 1 0.59 ± 0.11, group 2 0.64 ± 0.14. Skill mix - no statistically significant changes found during intervention period on all 4 core measures for either group.
Park et al. (2012)	Non-ICU units in participating hospitals; 2005	Examined the relationship between RN staffing and FTR carried with patient turnover levels.	Descriptive correlational design Hospitals: 42 Units: 759	RN HPPD; Data set analysis (UHC, AHA Annual Survey of Hospitals)	FTR defined as mortality in surgical patients preceded by a hospital acquired complication (pneumonia, deep vein thrombosis or pulmonary embolism, sepsis, acute renal failure, shock or cardiac arrest, and gastrointestinal haemorrhage or acute ulcer).	RN HPPD mean on adult non-ICUs 6.74 ± 1.4 and adult ICUs 15.52 ± 2.03. After adjusting for non-RN staffing, patient case mix, and hospital technological complexity, higher RN staffing levels on non-ICUs were significantly associated with lower rates of FTR (- 0.027[0.052, 0.002] p < .036, see Model 2). A change of 43.1% was noted in the coefficients for RN staffing on non-ICUs before and after the adjustment. Unlike the results for non-ICUs, unadjusted effect of RN staffing on FTR in ICUs was statistically significant (-0.021 [0.038, 0.004] p< .018).

First author (Yr)	Setting; Timeframe	Aim	Study Design; No. of participants	Definition of skill mix; Tool/ Instrument	Patient outcomes	General Findings
Patrician et al. (2011)	Military hospitals; 2003-2006	The association between nurse staffing and adverse events at the shift level.	Longitudinal, correlational Hospitals: 13 Units: 56	Proportion of hours worked by each skill level of staff (RN, LPN, and unlicensed provider) during a shift. Shift interval was standardized to an 8-hour period; Data set analysis (MilNOD)	1) Patient falls (patient's un-planned descent to the floor, with or without injury) and 2) medication errors (deviation from the physician's documented order committed by a nurse)	The average skill mix of RNs on medical-surgical wards was 51%, step-down wards was 58% and critical care was 77%. Greater proportion of RNs significantly associated with fewer falls and less medication errors in medical-surgical and critical care units but not in step-down units. Falls: skill mix (10% decrease), % RN OR 1.11 med/surg & OR 1.20 critical care. Skill mix (10% decrease) % LPN OR 1.08. Falls w/injury: Skill mix (10% decrease) % RN OR 1.30 med/surg, OR 1.36 critical care. Medication error: skill mix 10% decrease % RN OR 1.13 med/surg, OR 1.17 cc. Skill mix (10% decrease) % LPN OR 1.10 med/surg.
Patrician et al. (2016)	Medical-surgical, stepdown, and critical care units in military hospitals; 2003 - 2006	Evaluate the effects of nurse staffing on HAPI development, data on nursing care HPPD, nursing skills mix, patient turnover and patient acuity.	Longitudinal, correlational Hospitals: 13 Units: 56 n=1,643	% RN, % LPN, % NA; Data set analysis (MilNOD)	1) HAPI development. HAPI was defined as a pressure injury of Stage 2 or greater that was not present on admission	RN skill mix was NOT associated with HAPI (lower levels of total nursing care HPPD, RN nursing care HPPD, and LPN nursing care HPPD were associated with HAPI)
Paulson (2004)	ED unit in military hospital; Jan 1997 - Apr 1998	Compared the wait time and number of patients who LWBS between triage system that uses nurses verses UAP.	Comparative descriptive, retrospective chart review	2 triage systems using nurses (including LPN, nurses with associate's degree, nurses with baccalaureate) versus UAP; Data set analysis (ED reports); chart reviews using investigator designed data collection sheet	Wait time of patients who LWBS	The average difference in patient wait time was 73 minutes (57% decrease; p<0.000) and there was an 85% decrease in the number of patients who LWBS.
Person et al. (2004)	Cooperative Cardiovascular Project linked hospitals; 1994 - 1995	Assess the association of nursing staff with in-hospital mortality for patients with acute MI.	Descriptive correlational design Hospitals: 4401 n=118,940	Ratio of full-time equivalent RNs to average daily census and the ratio of FTE LPNs to average daily census; Data set analysis (CCP dataset, CMS administrative data)	1) In-hospital mortality for patients with acute MI	Skill mix mean ratio was 14.6±30.6 (quartile 4 19.8 ± 38.7, quartile 3 16.3 ±25.2, quartile 2 12.3 ± 26.6 and quartile 1 10.0 ± 29.4). Higher RN staffing associated with patients less likely to die: OR (95% CI) quartiles 4, 3, and 2 vs quartile 1 0.91 (0.86–0.97), 0.94 (0.88 –1.00), and 0.96 (0.90–1.02), respectively. Higher LPN staffing: patients = more likely to die in-hospital; ORs (95% CI) quartiles 4, 3, and 2 vs quartile 1 were 1.07 (1.00 –1.15), 1.02 (0.96 – 1.09), and 1.00 (0.94 –1.07) respectively.

First author (Yr)	Setting; Timeframe	Aim	Study Design; No. of participants	Definition of skill mix; Tool/ Instrument	Patient outcomes	General Findings
Pitkaaho et al. (2015)	Acute care university hospital; 2008	Analyse relationships between nurse staffing and patients' LOS.	Time series. Retrospective longitudinal design; Hospitals: 1 Units: 20 n=35,306 patient episodes	Average proportion of RNs; Review of patient episodes and nursing related administrative information	LOS	Skill mix RNs proportions of 65–80% was conducive to short LOS and predicted a 66% likelihood of short length of stay. Higher (80-100%) and lower (47-65%) percentages of RNs predicted lower likelihood of short LOS.
Potter et al. (2003)	Acute inpatient units at a denominational hospital; Feb 2000 - Jan 2001	To determine baseline values of patient outcome measures and the relationship of nurse staffing.	Prospective, correlational design Hospitals: 1 Units: 32	Average % of RN and UAP hours of direct care; Administrative records of adverse events, inpatient self-reports, post discharge patient satisfaction	1) patient falls, 2) medication errors	The yearly average % of nursing care provided by RNs on day shift was 55.5% ± 7.8% and the average number of hours of direct patient care provided by RNs on day shift was 3.0 ± 6.0. No findings relating to skill mix. Higher number of care hours, irrespective of category, associated with fewer falls.
Roche et al. (2012)	Wards across public hospitals; 2004-2006 (2.5 years)	Examine the relationship between staffing, skill-mix and incidence of NSOs.	Longitudinal, descriptive Hospitals: 2 Units: 14	RN hours as a % of total nursing hours; Data set analysis (AR-DRG, ICD-10-AM); ward-episode data; nursing payroll data)	1) CNS complications, 2) decubitus, 3) FTR; (death following sepsis, pneumonia, GI bleeding, or shock), 4) ulcer/GI bleeding , 5) pneumonia, 6) sepsis, 7) UTI	Increase of 10% in proportion of hours worked by RNs linked to decrease in NSO rates by 11% pneumonia, 15% sepsis, 19% decubitus, 27% FTR, 34% UTI, 37% GI bleed, and 45% CNS complications.
Schneider and Geraedts (2016), Germany	Acute care hospitals; Jan - Mar 2010	Association between nurse and physician staffing and the incidence of HAPU.	Cross-sectional Hospitals: 720 1st quarter of 2010 n= 716,281 2nd quarter n=757,665	% of nurses with at least 3 years of training to total nursing staff (general nurses, paediatric nurses, and geriatric nurses), comparing 2010 to 2012; Secondary data from the structured quality reports of hospitals (SQR), supplemented with data from the German Hospital Directory (GHD).	Standardised incidence ratios of HAPU.	The median nursing skill mix was 95.68 (IQR 5.43) in 2010 and 95.69 (IQR 5.89) in 2012. Estimated coefficients suggest that a 10% increase in the proportion of nurses with at least 3 years of training to total nursing staff was associated with a decrease in HAPU observed/ expected ratios of 0.12–0.15 points. (multivariate) observed/ expected ratio pressure ulcers stage II-IV 2010 -0.015 (-0.025;-0.004) p=0.008, observed/ expected ratio pressure ulcers stage I-IV 2010 -0.014 (-0.024;-0.004) p=0.006, observed/ expected ratio pressure ulcers stage I-IV 2012 -0.012 (-0.025;-0.004) p=0.019

First author (Yr)	Setting; Timeframe	Aim	Study Design; No. of participants	Definition of skill mix; Tool/ Instrument	Patient outcomes	General Findings
Schreuders et al. (2015)	High intensity, general medical and general surgical units at metropolitan, tertiary hospitals; 2004 - 2008	Examine the impact of nurse staffing on impatient complications across different unit types.	Retrospective longitudinal hospitalization-level study<br>Hospitals: 3<br>Units: 58<br>n=256,984	Proportion of total nursing hours worked by RNs. Separated into 3 categories based on % of total nursing care hours provided by RNs: low (RNs provided <74% of hours), medium (RNs provided ≥74% and <83% of hours) and high (RNs provided ≥83% of hours);<br>Data set analysis (WA Department of Health administrative data collections)	Inpatient complications: surgical wound infection; UTI; pressure ulcer; pneumonia; DVT and shock or MI; FTR (death following pneumonia, shock or cardiac arrest, upper GI bleeding, sepsis or DVT) and 30-day mortality.	The average RN skill mix for high-intensity units was 86.5 ± 8.94 and for low intensity units was 75.8 ± 11.15. Direction of the association between nurse staffing and patient complications was not consistent. Surgical wound infection and urinary tract infection were the only complications that consistently decreased with improved staffing levels. Surgical wound infection (none of the length of stay on a high-intensity unit) NHpPD & Low RN% OR (95% CI) OR 1. 29 (1.22–1.37) p<0 001; Surgical wound infection (whole length of stay on a high-intensity unit) NHpPD & Low RN% 0. 92 (0.86–0.99) p=0. 025; Mid RN% 0.85 (0.79–0.92) p<0 001; UTI mid RN% 0.85 (0. 79–0.92) p<0.001, high RN % 0.87 (0.82–0.93) p<0.001, Surgical wound infection (both high- and low-intensity units) low RN % 0.91 (0.83–0.99) p=0.029; mid RN % 0.90 (0.85–0.95) p<0.001, UTI (both) mid RN%0. 93 (0.89–0.96) p<0.001 0.92 (0.88–0.97) p=0.001
Seago et al. (2006)	University teaching hospital; Adult medical and surgical nursing units;<br>1999 - 2002	Compare the relationship between nursing staffing and positive patient outcomes.	Longitudinal, retrospective repeated measures design<br>Hospital: 1<br>Units: 3	Proportion of RN hours divided by total hours, RNHPPD =Total RN hours divided by total patient days;<br>Data set analysis (internal hospital databases)	1) FTR from medication error, and 2) FTR from decubitus	The mean skill mix per month for unit A was 0.75 (0.02), unit B 0.96 (0.04) and unit C 0.72 (0.02). There was no difference in FTR from medication error or FTR from decubitus in relation to skill mix. There was an increase in FTR from medication error as the non-RN (Other) hours of care per patient day increased.
Sochalski et al. (2008)	Acute care general hospitals; 1993 - 2001	To determine whether increases in medical-surgical licensed nursing staff levels are associated with improvements in patient outcomes	Cross-sectional<br>Acute MI n=348,720<br>FTR n=109,066	RN and RN / LVN nurse staffing;<br>Data set analysis (OSHPD, Centers for Medicare and Medicaid Services, Census Bureau data)	1) 30-day Acute MI mortality and 2) surgical FTR	Medical surgical RN hours/patient day, baseline year 4.29 (3.51 - 5.00) and annual rate of change 1.2 (-6.66 - 9.6). No findings relating to skill mix (fixed effect models). An increase in RN and RN - LVN hours per patient day was not significantly associated with reductions in acute MI mortality or FTR.
Sovie et al. (2001)	University teaching hospitals;<br>Jul 1997 - Jun 1998	Describe the effects of nursing structure and processes on selected patient outcomes	Descriptive, longitudinal<br>Hospitals: 29	HPPD for all staff, for RN, UAP and Other;<br>Data set analysis (MECON-PEERx), assessment instrument and interviews with Chief Nurse	1) Fall rate, 2) pressure ulcer, and 3) UTI	RN HPPD Hospital nursing departments 8.09-8.45, medical units 5.10-5.52 and surgical units 5.15-5.18. Fall rate declines as number of RN HPPD increases (F=11.73, P= .002).

First author (Yr)	Setting; Timeframe	Aim	Study Design; No. of participants	Definition of skill mix; Tool/ Instrument	Patient outcomes	General Findings
				Executives; MPOP Questionnaire; 4-item subscale from the Quality of Employment Survey; patient satisfaction data using institutional tools and procedures		
Staggs et al. (2014)	Units in NDNQI participating hospitals; 2011	Explore non-linear associations between unassisted fall rates and level of RN and non-RN staffing.	Cross-sectional Hospitals: 1361 87 544 unit-months of data	RN HPPD (sum of nursing care hours provided by RNs during the month divided by the sum of the unit's patient days for the month) & Non-RN HPPD (hours provided by LPNs and assistive personnel); Data set analysis (NDNQI)	Monthly unit-level data on inpatient falls	The HPPD by ward, step-down RN 7.4 and non-RN 2.7, Medical RN 5.8 and non-RN 2.8, Medical-surgical RN 5.8 and non-RN 2.8, Surgical RN 6.0 and non-RN 2.8 and rehabilitation RN 4.5 and non-RN 3.5. For all unit types except rehabilitation, higher non-RN staffing was associated with higher rates of unassisted falls. Medical -surgical - estimated average fall rate decreased by 2% (95% CI: 0–3%) per additional RN HPPD.
Staggs et al. (2012)	Nursing units in hospitals participating in NDNQI; Oct 2009 -Sep 2010	To explore hospital and nursing unit characteristics as predictors of unassisted fall rates.	Longitudinal Hospitals: 248 Units: 1504	Proportion of the month's total nursing care hours provided by RNs; Data set analysis (AHA annual survey data	Unassisted fall rate (no nursing staff present to mitigate the effects of a fall)	The mean skill mix was 0.72 ± 0.14. Increase of 0.14 (1 SD) in proportion of nursing care hours provided by RNs is associated with an estimated 4.0% average decrease in the rate of unassisted falls.
Staggs et al. (2016)	Medical, surgical, and medical-surgical units in hospitals participating in NDNQI; Oct 2006 - Dec 2010	Examining whether nurse staffing relative to a unit's long-term average is associated with restraint use	Longitudinal Units: 3101	Proportion of nursing hours provided by RNs, units reporting no restraint use compare to units reporting restraints; Data set analysis (NDNQI)	1) Restraint use for any purpose reported for at least one patient on the unit in quarterly survey. 2) Restraint use for the purpose of fall prevention. 3) Each unit's overall restraint prevalence (proportion of total patients assessed during the study who were restrained) and fall prevention restraint prevalence (proportion of patients assessed	The mean skill mix was 65.3 ± 9.4, units reporting no restraint use 66.4 ± 10.6 and units using restraint 66.3± 10.6. Strong negative correlation between nursing skill mix and physical restraint use. Statistically significant effects of skill mix category on odds of any restraint (p<0.001) and odds of fall prevention restraint (p=0.035). Compared to quarters with average skill mix, adjusted odds of any restraint use 11% (95% CI 1.04–1.19) and 18% (95% CI 1.08–1.29) higher, respectively, for quarters with low and very low skill mix. Odds of fall prevention restraint were 9% (95% CI1.00–1.19) and 16% (95% CI 1.03–1.29) higher, respectively, for quarters with low and very low skill mix. Mean skill mix was a significant inverse predictor for restraints (OR 0.87, 95% CI 0.82–0.92) and fall prevention restraint 0.93 (95% CI 0.88–0.98).

First author (Yr)	Setting; Timeframe	Aim	Study Design; No. of participants	Definition of skill mix; Tool/ Instrument	Patient outcomes	General Findings
					who were restrained for fall prevention)	
Tourangeau et al. (2002)	Acute care hospitals; patients with a diagnosis of MI, stroke, pneumonia or septicaemia; 1998 - 1999	Investigating the effects that nursing care has on commonly recognised quality of care outcomes	Retrospective design<br>Hospitals: 75<br>Patient records (N = 46,941) and nurse staffing responses (N = 3,995)	RN inpatient earned hours proportionate to other inpatient nursing staff earned hours;<br>Review of patient records; Ontario Registered Nurse Survey of Hospital Characteristics	1) 30 day risk-adjusted mortality rate	The mean skill mix was 0.75 (±0.11). 10% increase in RNs associated with 5 fewer patient deaths per 1000 discharges. Skill mix: parameter estimate= -0.0489 F= -1.97, P= 0.04
Twigg et al. (2016)	Acute care metropolitan hospitals;<br>Jan 2006 - Dec 2010	To assess the impact of adding AIN to acute care hospital ward nurse staffing on adverse patient outcomes.	Descriptive cohort study (pre-test/post-test control group design)<br>Hospitals: 11<br>N= 256,302 (Pre-test n=125,762 & post-test n= 130,540)	NHPPD ratings for the AIN wards compared to the non-AIN wards;<br>Data set analysis (WA Data Linkage Unit)	1) In-hospital 30-day mortality, 2) FTR, 3) UTI, 4) pressure injury,<br>5) pneumonia, 6) sepsis and 7) falls with injury	The median was 87% RNs (range of 51%-100%) for the non-AIN wards and median of 77% (range 40-98%) for AIN wards. Skill mix (RN/EN) of the 2 ward types remained at similar levels across the pre-test and post-test periods. Pre-test/post-test analysis showed 3 significant increases in adverse outcomes on the AIN wards (FTR, UTI, falls with injury): FTR Difference (Observed-expected) 68 p=0.018, UTI 204 p=0.017, falls 165 p< 0.001; decreased mortality -108 p=0.024
Twigg et al. (2012)	Multi-day wards in acute care hospitals;<br>Oct 2002 - Jun 2004	To explore the association between skill mix and NSOs following implementation of nursing HPPD.	Retrospective, longitudinal analysis of administrative data (interrupted time series)<br>Hospitals: 3 (52 units)<br>Patient records (N = 103,330) and nurse staffing records (N = 73,770)	Proportion of total nurse hours provided by RNs (in %);<br>Data set analysis (hospital morbidity data)	NSOs (CNS complications; wound infections; pulmonary failure; UTI; pressure ulcer; pneumonia; DVTs; ulcer/ gastritis/ upper gastro-intestinal bleed; sepsis; physiological/ metabolic derangement; shock/ cardiac arrest; mortality; FTR; LOS)	The mean skill mix for Hospital 1 was 88.5 (range: 87.5-89.8), hospital 2 was 81.5 (range: 78.5-83.5) and hospital 3 84.1 (79.9-88.7). Hospital 1 rate of pneumonia increased significantly with each percentage-point increased in skill mix 1.16* (1.01, 1.33) P <0.05; Hospital 2 pneumonia 0.90 (0.85, 0.97) P< 0.01, DVT 0.81 (0.70, 0.93) P< 0.01, shock/cardiac arrest 0.73 (0.60, 0.88) P<0.001, and FTR 0.88 (0.79, 0.99) P <0.05 decreased significantly, UTI increased 1.07 (1.02, 1.13) P< 0.01, Hospital 3 pressure ulcer 0.91 (0.86, 0.97) P< 0.01, gastritis and upper GI bleeds decreased 0.95 (0.90, 0.99) P <0.05, rate of shock/cardiac arrest in medical patients increased significantly 1.10 (1.00, 1.22) P <0.05

First author (Yr)	Setting; Timeframe	Aim	Study Design; No. of participants	Definition of skill mix; Tool/ Instrument	Patient outcomes	General Findings
Tzeng, et al. (2011)	Acute care hospitals; **Timeframe not supplied**	To determine 2 nursing staff indicators (FTEs per 100 discharge and % of RN FTEs by total nursing personnel FTEs) on hospital-acquired injurious fall rates.	Retrospective analysis Hospitals: 244	Precent of RN FTEs by total nursing personnel FTEs; Data set analysis (FY2007 data, CMS Hospital Consumer Assessment of Healthcare Providers and Systems data web site, AHA Annual Survey Database), CMS Case Mix Index.	Hospital-acquired injurious fall rates	The mean percent of RN FTEs by total nursing personnel FTEs was 74% ± 0.1. Higher % of RN FTEs by total nursing personnel FTEs did not result decreased injurious fall rates.
Unruh (2003)	Acute care hospitals; 1991-1997	To examine the relationship of licensed nursing staff with patient adverse events in hospital	Retrospective, longitudinal analysis Hospitals: 211	Proportion of licensed nurses /total nursing staff; Data set analysis (PDH and AHA data - nursing and hospital characteristics; PHC4 – patients)	Yearly 1) iatrogenic lung collapse, 2) pressure sores, 3) falls, 4) pneumonia, 5) post-treatment infections, and 6) UTIs	The mean proportion of licensed staff was 0.847. Hospitals with more licensed nurses had significantly lower incidences of atelectasis - 1.5%, decubiti -2% , falls -3%, and urinary tract infections -<1%, but higher rates of pneumonia +<1%. Hospitals with a greater proportion of licensed nurses /total nursing staff had significantly lower rates of decubiti - 2% and pneumonia - <1%. Licensed nurse/total nursing staff had no significant association with the other adverse events with the exception of a positive relationship to falls.
Unruh et al (2012)	Acute care hospitals; 1996 - 2004	To examine the relationship between changes in RN staffing and patient safety	Retrospective, longitudinal analysis Hospitals: 124	Two measures of RN staffing -RN FTEs and RN per adjusted patient day (RN/APD); Data set analysis (AHCA - Florida Hospital In-patient data; AHA annual survey data; Medicare Public Use Files)	Patient safety measures: 1) decubitus ulcer, 2) FTR, 3) selected infections due to medical care, and 4) postoperative sepsis	Changes over time in RN FTEs (increased) negatively related to changes over time in FTR - 2.881 P <0.05 and positively related to changes over time in selected infections 2.195 P<0.05. A change in RN/APD over time was negatively related to changes over time in selected infections -2.106 P <0.05 and postoperative sepsis -2.630 P <0.05.
Yang et al. (2015)	Respiratory care centre; 2006 - 2016	To examine the impact of application of different nursing staffing models on patient safety, quality of care ad nursing costs.	Retrospective cohort study n=667	Proportion of RNs= % of RNs to total nursing staff (RNs and nurse aides); 3 mixed models of nursing staffing, % of nurses compared with nurse aides was 76%, 100% and 92%; Data set analysis (nursing care quality and patient safety departments, patient records; hospital accounting office)	Rates of: pressure ulcers; UTIs, respiratory tract and bloodstream infections; medication errors; unplanned endotracheal tube extubation; ventilator weaning	76% RNs group made fewer medication errors than the 100% RNs group (OR:0.37, 95% CI 0.14–0.88) ; the 76% (OR: 4.36, 95% CI 2.29–8.92)and 92% (OR:3.12, 95% CI 1.42–7.23)RNs groups had a higher rate of UTIs; the 92% RNs group had a lower rate of bloodstream infections (OR:0.33, 95% CI 0.14–0.71) ; the 76% RNs group had a lower rate of ventilator weaning (OR:0.19, 95% 0.04–0.86)

First author (Yr)	Setting; Timeframe	Aim	Study Design; No. of participants	Definition of skill mix; Tool/ Instrument	Patient outcomes	General Findings
					and mortality, length of ventilator use; LOS	
Yang (2003)	Medical-surgical units in a tertiary care centre; 2000 (fiscal year)	Examine the relationship between hospital nurse staffing and patient NSOs	Retrospective, descriptive correlational design Units: 21 n=29,424	Ratio of RNs to average patient census -no. of RNs in direct patient care divided by number of the average occupied inpatient census at the unit level. Daily average hours of care provided by RNs, Skill mix N1, N2, and N3 Data set analysis (NQIC; hospital generated monthly statistical reports)	Rates of: patient falls, pressure ulcers, respiratory tract infections and UTIs.	The daily average hours of care pre patient were 2.9 (range:2.57-3.55). Skill mix of RNs sampled by category as N1, (N - 225, 64.84%), N2, (N =57, 16.43%) and N3 (N=65,18.73%). No patient outcomes relating to skill mix. Ratio of RNs to patient census negatively correlated to patient falls (r = -.483, p <.05), UTI (r - .397, p< .10) and complaints (r = -.440, p< .05).

Abbreviations: ACS=acute coronary syndrome; AHRQ=Agency for Healthcare Research and Quality; AHA=American Hospital Association; AIN=Assistants in Nursing; AR-DRG=Australian Refined Diagnostic Related Group; BG=blood glucose; CI=confidence interval; CHF=congestive heart failure; CMI=Case-mix Index; CNS=Clinical Nurse Specialist; CNS=Central Nervous System; COPD=chronic obstructive pulmonary disease; CCP= Cooperative Cardiovascular Project; DRG=diagnosis-related group; DVT=Deep vein thrombosis; ED=Emergency department; EN=Enrolled Nurse; FIM=Functional Independence Measure; FTE=full time equivalent; FTR=Failure to rescue; GI=gastro intestinal; HADR=Hospital Annual Disclosure Report; HAPI= Hospital acquired pressure injuries; HAPU=Hospital acquired pressure ulcers; HCAHPS= Hospital Consumer Assessment of Healthcare Providers and Systems; HCUP=Healthcare Cost and Utilization Project; HCSW=Healthcare Support Workers; HIRA=Health Insurance Review and Assessment; HPPD= hours per patient day; ICD-10-AM=International Classification of Diseases; ICU=Intensive care unit; LOS=Length of stay; LPN= Licensed practical nurse; LVN=licensed vocational nurse; LWBS=left without being seen; MPOP=Management Practices and Organizational Processes Questionnaire; MilNOD=Military Nursing Outcomes Database; MI=myocardial infraction; N=number; NA=Nursing assistant; NAA=nurse-administered analgesia; NDNQI=National Database of Nursing Quality Indicators; NHI=National Health Insurance; NIS=Nationwide Inpatient Sample; NMDS=National Minimum Dataset; NQIC= Nursing Quality Improvement Committee; NSO=nurse sensitive outcomes; NWD=Nursing Workforce Dataset; NWI-R=Nursing Work Index; OR=odds ratio; OSHPD=Office of Statewide Health Planning and Development; PDH= Pennsylvania Department of Health; PES=Practice Environment Scale; PHC4= Pennsylvania Health Care Cost Containment Council; RPN=registered practical nurse; RN= Registered Nurse; SID=State Inpatient Databases; TEN=Trainee Enrolled Nurses; THG=Total number of episodes of hypoglycaemia; TotHPD=Total hours per day; UAP: unlicensed assistive personnel; UAPU=Unit acquired pressure ulcer; UHC=University HealthSystems Consortium; UNA=Unlicensed nursing assistants; UTI=urinary tract infection; VA= Veteran Affairs; %=Percent;
